# Transepithelial Effect of Probiotics in a Novel Model of Gut Lumen to Nerve Signaling

**DOI:** 10.3390/nu14224856

**Published:** 2022-11-17

**Authors:** John E. Piletz, Jason Cooper, Kevin Chidester, Kyle Erson, Sydney Melton, Anthony Osemeka, Megan Patterson, Kyndall Strickland, Jing Xuan Wan, Kaitlin Williams

**Affiliations:** 1 Office of Global Education, Mississippi College, Clinton, MS 39058, USA; 2 Department of Biology, Mississippi College, Clinton, MS 39058, USA

**Keywords:** enteric, nervous system, cholinergic, probiotics, gut, brain, commensal, *Caco2*, *SH-SY5Y*, transwell, neurites, gut brain axis

## Abstract

Recent studies have shown that the gut microbiome changes brain function, behavior, and psychiatric and neurological disorders. The Gut–Brain Axis (GBA) provides a neuronal pathway to explain this. But exactly how do commensal bacteria signal through the epithelial layer of the large intestine to activate GBA nerve afferents? An in vitro model is described. We differentiated two human cell lines: *Caco2Bbe1* into mature epithelium on 0.4-micron filters and then *SH-SY5Y* into mature neurons in 24-well plates. These were co-cultured by placing the epithelium-laden filters 1 mm above the neurons. Twenty-four hours later they were tri-cultured by apical addition of 10^7^ *Lactobacillus rhamnosus* or *Lactobacillus fermentum* which settled on the epithelium. Alone, the *Caco2bbe1* cells stimulated neurite outgrowth in underlying *SH-SY5Y*. Beyond this, the lactobacilli were well tolerated and stimulated further neurite outgrowth by 24 h post-treatment, though not passing through the filters. The results provide face validity for a first-of-kind model of transepithelial intestinal lumen-to nerve signaling. The model displays the tight junctional barrier characteristics found in the large intestine while at the same time translating stimulatory signals from the bacteria through epithelial cells to attracted neurons. The model is easy to set-up with components widely available.

## 1. Introduction

The old concept of an enteric nervous system (ENS) that functions independently from the central nervous system (CNS) [1], is a dogma fallen by the wayside [2]. A major paradigm shift has occurred. We now know that a branch of the ENS continuously monitors the gut and provides moment-by-moment communication to/from the brain [3]. This branch is called the Gut–Brain Axis [4]. Although the ENS can coordinate peristaltic movements and gastric secretions without the need for input from the CNS [1], the information transmitted through the GBA to the brain appears essential for metabolism and mental health [5]. Closely intertwined with this new paradigm are studies linking the gut microbiome to previously thought to be brain-exclusive diseases [6]. These include autism spectrum disorder, amyotrophic lateral sclerosis, transmissible spongiform encephalopathies, Parkinson’s disease, Alzheimer’s disease, and major depression [2]. There is also evidence that our gut microbiomes explain aspects of human temperament and personality [7]. In short, the ENS is a cooperative second brain and our gut microbiomes have achieved accessory organ status [8].

The physiological system proposed to integrate information from the microbiome is the GBA [6]. The GBA has a network of nerves spread throughout the GI tract to funnel information through vagal and splenic nerves to/from the brain [9]. Their point of interface is believed to be cholinergic afferents and neuroendocrine cells underlying the epithelial lining of the gut [10]. There are immunoreactive cells located in the interstitial space as well [11], so the exact boundaries of the GBA remain poorly defined [4,12]. That the GBA not only channels information but exerts its own physiological consequences through feedback setpoints akin to the autonomic nervous system, is implied but not fully understood.

We are not the first to model the interstitial space under the gut epithelium [13,14,15]. However, models to date have been aimed elsewhere. Namely, they have aimed at signals from nerve-to-epithelium, or lumen-to-immunocytes, or lumen-to-neuroenteroendocrine cells [11]. Some have shown in hindsight to be models of gastrointestinal (GI) cancer cell interactions [13,16]. Perhaps the closest model uses induced pluripotent stem cells (iPSCs) in the form of miniature gut-like organoids [17]. Their theoretical potential is great because iPSCs differentiate into any number of cell types. An elegant model called “iPSC gut-on-a chip” has been reported [15], complete with variations in hydrogels [18] or fitted with fluidic controllers and custom-made biosensors [19,20]. One “iPSC gut-on-a chip” is being marketed by a biotech company (https://emulatebio.com, accessed on 7 November 2022, Boston, MA, USA) [21]. Yet, we see a shortcoming in them because there are no reports of neuroactive cells acting in these models.

A new model system is presented based on two well-known cell lines, *Caco2Bbe1* and *SH-SY5Y*. We describe how to convert these lines into their normal epithelium and neuronal phenotypes, respectively, and then juxtapose them 1 mm apart by a filter. The idea came from an earlier paper [16] in which we used the same lines to demonstrate the anti-neuroblastoma capacity of cruciferous vegetable extracts when transepithelially applied. That study fell into the field of nutraceutical anti-cancer modeling. By pre-converting *SH-SY5Y* cells into ‘normal’ neurons before co-culturing with mature *Caco2bbe1′s*, we now have a different model. Namely, one suited to the study of normal lumen-to-nerve signaling as in the human intestine.

Our model is a first-of-kind. The model is in alpha version stage. Future improvements should be forthcoming. There is no reason why our two cell lines, once transitioned as described in this paper to their mature states, could not become a “gut-on-a-chip”, made with hydrogels, and/or fitted with fluidic controls [20]. Also, the reverse flow of information (from neurons to epithelium to luminal microbiota) stands to be another line of investigation. Some knowledge about the reverse flow of information already exists using gut mucosal explants containing intact neurons co-cultured with *Caco-2* cells [22,23]. Adding pathological bacteria also stands for future study of Chron’s disease or inflammatory bowel disorders. Finally, analytical chemistry needs are applied in future studies of our model to identify candidate neurotransmitters, such as acetylcholine, agmatine, and serotonin [24,25].

## 2. Materials and Methods

### 2.1. Cells, Media, and Culturing Set-Up

Purchased from ATCC (Manassas, VA, USA), the human neuroblastoma cell line, *SH-SY5Y*, and the human colon adenocarcinoma cell line, *Caco2Bbe1,* arrived in cryovials on dry ice. Upon thawing, a common media was identified to differentiate both, made with a base of HyClone^TM^ media (Cytiva Life Sci. Cat. #SH30023.01) containing DMEM/F12 Ham’s media (1:1) supplemented with 2.5 mM L-glutamine, 15 mM HEPES sodium bicarbonate, and phenol red, purchased in a 500 mL bottle. To make complete media—used to grow the cells—we added 55 mL of pre-filtered Fetal Bovine Serum (FBS) using a Millipore 0.1 micron PES filter, and 5.5 mg of human transferrin (Sigma-Aldrich, St. Louis, MO, USA). We also added 5 mL penicillin/streptomycin concentrate (55 I.U./mL, Fisher #15-140-122) except in experiments using live probiotics. One of our previous publications [26] describes the importance of pre-identifying suitable lots of FBS and the need to slowly thaw it. Just before use, aliquots (40 mL) of complete media were warmed in a water bath (37 °C). A humidified incubator (NuAire model 4750, Plymouth, MN, USA) was used to grow the cells, at 37 °C with gas flow at 20 psi of filtered 5% CO_2_. Changing the intake gas filter periodically was important since not doing so led to detachment of the *Caco2Bbe1* cells (personal experience). Also, media stored at 5 °C was used no more than 9 months after the manufacturing date due to the instability of L-glutamine. The plasticware for the co-culture model includes sterile inserts (Millicell^®^-PCF, 0.4-micron pores, 12 mm diameter, Merck Millipore, Cork, IRL) and at least two standard 24-well plates.

### 2.2. Maintaining SH-SY5Y Parent Line

Maintenance of the *SH-SY5Y* neuroblastoma phenotype was done in T75 flasks (75 mm size) filled with 16 mL of complete media. Passaging occurred every 6 days. This involved first discarding spent media along with washes of debris and floating cells. Only attached cells were carried forward. These were detached for passaging by 2-min digestion (at 1.6 mL per flask) with 0.25% porcine trypsin-EDTA (Sigma-Aldrich). Trypsin digestion was terminated by 10-fold dilution with complete media followed by trituration 10 times with a wide bore 10 mL pipette. Only one-fourth of the resuspended volume was taken forward each generation in a parent flask by diluting in complete media, 1:4. About 3 × 10^5^ viable cells were thus replated each generation. By the 3rd generation, there was ample surplus for experimental purposes (at least 9 × 10^5^ viable cells every 6 days). The *SH-SY5Y* line was allowed to a 50th generation before we restarted from the source frozen plugs that had been prepared after the original shipment from ATCC. 

### 2.3. Maintaining Caco2Bbe1 Parent Line

*Caco2Bbe1* parent flasks were grown in like manner except for a few accommodations because *Caco2Bbe1′s* grow faster and achieve more (90–100%) confluence over the same 6 days period. To detach them for passaging required 12 min in trypsin-EDTA. Another distinction was that after dilution, trituration, and washing, just one-fifth (rather than one-fourth) of suspended *Caco2Bbe1′s* were carried forward to the next generation (i.e., 1:5 dilution in new media in a new flask) [16]. Experiments with the *Caco2Bbe1* line were also held to 50 generations to avoid mutations.

*What to watch for?* Based on cell numbers, the *Caco2Bbe1* line multiplies faster than *SH-SY5Y*. Evidenced over 6 days, the *SH-SY5Y* flasks typically reached 50% confluency and quadrupling of cell number, compared to the *Caco2Bbe1* flasks which typically reached ≥90% confluency and quintupling of cell number. Secondly, the *Caco2Bbe1* line displayed almost no floater cells, whereas the *SH-SY5Y* line had many floaters [27]. These floaters arise by budding off from *SH-SY5Y* somas. The C*aco2BBe1* line attaches so firmly that 12 min was needed in trypsin-EDTA. This may impart a slightly lower percent viability to the harvested epithelial cells (e.g., trypan blue staining showed about 70% viability versus 95% viability for C*aco2BBe1* compared to *SH-SY5Y* each harvesting). Another distinguishing factor was the *Caco2BBe1* cells caused media to change color from pink to gold, indicative of progressive acidity over 6 days to about pH 6.0. This reportedly [28] owes to the secretion of gastric acid and mucus from the microvilli of *Caco2Bbe1*. It was one reason we studied conditioned media (Figure S1). The *SH-SY5Y* line also needs to be monitored because it is prone to accumulating mutant epithelial phenotypes [27].

### 2.4. Direct Co-Culture of Caco2Bbe1 and SH-SY5Y

Prior to developing the full model (Figure S2), direct co-cultures were studied. For starters, *Caco2bbe1* and *SH-SY5Y* were combined in suspension and directly plated together in equal numbers in open wells. From these studies, it became clear that the two lines would grow well together and retain distinctive phenotypes over many days. But the neurites were obscure when directly co-cultured because cells tended to overlay each other. Therefore, a “swipe-and-wash” protocol was developed on mature *Caco2bbe1* cells before directly co-cultured (Figure S3). Swiping created a space wherein added *SH-SY5Y* cells could settle and develop adjacent to, but not overlaying, *Caco2bbe1′s.* Setting it up began with *Caco2Bbe1′s* plated at high density (1.1 × 10^5^ in 3 mL/well complete media) on multiple 6-well plates (Corning^®^ Costar^®^, flat bottom: Sigma-Aldrich, St. Louis, MO, USA). These were grown to confluency after 5 days in the incubator. The lawn of cells was then given a single swipe with the blunt end of a sterile p200 pipette tip. The detached cells were pipetted to waste over 6 washes of each well with sterile phosphate-buffered saline (PBS). The swipe zones covered an area averaging 3 mm wide in the middle of each well. Immediately thereafter, a fresh 3 mL media suspension of *SH-SY5Y* cells was added (*n* = 2000 *SH-SY5Y* cells per well). Three control groups were also set up with each experiment: (1) *n* = 2 wells swiped, washed, and refed complete media without cells; (2) *n* = 2 wells not swiped-and-washed but given fresh media with *SH-SY5Y cells*; (3) *n* = 2 wells swiped-and-washed given fresh media with C*aco2Bbe1 cells*. An indelible marker was used to mark the swiped boundaries from underneath the wells. A single shake was applied to allow the *SH-SY5Y’s* to uniformly disperse before settling. They were refed complete media every two days for 6 more days. After this, photographs were taken with a calibrated microscope at 4× magnification (Micron) to measure the distance the epithelial cells grew inward. Then, the wells were washed and stained with crystal violet dye (below). Microscopic images at 10× magnification were also saved to count the number of *SH-SY5Y* cells in each well. Microscopic images at 20× were saved to assess the average proximity of the *SH-SY5Y* cells relative to the *CacoBbe1* border. Other images were saved at 40× to trace the shapes of *SH-SY5Y* cells for determining neurite areas (below). 

### 2.5. Conditioned Media

The conditioned media in our paper derive from complete media given different lengths of exposure to different types of cells. There were two main variations. One was derived from the 24 mL replenishment of a 24-well monoculture plate of confluent *Caco2Bbe1*e’s, conditioning from day 9 to day 11 since the cells were plated. The other was derived from the 24 mL replenishment of a 24-well plate of highly dense *SH-SY5Y* monocultures, conditioning from day 9 to day 11 since the cells were plated. Media from these cells was removed and centrifuged (5 min, 500× *g*) into pellet. The cell-free supernatants were used immediately as conditioned media to refeed 3-day-old SH-SY5Y cells in a separate plate (described below). In like manner, we also studied: (1) *Caco2Bbe1*e-conditioned media from wells that had been swiped and washed, (2) *Caco2Bbe1*e-conditioned (unswiped) media from below the inserts on day 11 before the *SH-SY5Y* cells were added, and (3) the media from a 24-well plate of *SH-SY5Y* monocultures plated just 1 day prior to collection (starting at *n* = 2000 cells/well). The test for all conditioned media came when replacing the media of growing 3-day-old attached SH-SY5Y wells. Endpoints came after 5 additional days of culture in the incubator. The sequence of events testing conditioned media added basally to the model is shown in Supplementary Figure S1. Cells were then washed and stained with crystal violet dye. Microphotographs were used to trace the shapes of cells and neurites. Cellometry was done to count the *SH-SY5Y* cells in each well (procedures below).

### 2.6. New Insert Co-Culture Model

By way of overview, our insert co-culture model is constructed in the following manner. *Caco2Bbe1* cells are plated at high density on filter inserts. On their 7th day of growth, a separate plating of lower-density *SH-SY5Y* cells occurs in a separate 24-well, flat-bottom plate. Both lines are grown separately for 5 more days. The neuroblastoma cells are then treated with 10 µM all-trans retinoic acid in serum-free media (RA; #MP021902696, MP Biomedical), which induces long neurites over the next 2 days. Meanwhile, the 14-day-old epithelial *Caco2Bbe1* cells on the filter inserts will have achieved full maturity with high transepithelial electrical resistance (TEER) indicative of tight junctions. These are then given low-serum media and transplanted atop the neurons. These are bathed from below in the same media that had induced *SH-SY5Y* neurites. In this fashion, the two cell lines are allowed to acclimate for 15 h in the incubator. Thereafter, any number of experiments can be performed over 56 h. Beyond that time, signs of cellular fatigue limit the utility of the model. A graphical depiction of how the model is prepared is shown in Supplementary Figure S2.

Step-1:  Making *Caco2Bbe1* into epithelium

Prior to seeding *Caco2Bbe1* cells, the inserts needed preparation. This is done by filling each of the wells in a 24-well plate with 0.5 mL complete media, placing inserts into each well, and placing the entire plates in the humidified incubator for 10 min. Nothing but air is within the upper chambers of the inserts, but the filters become wet from underneath. While this is happening, the *Caco-2Bbe1* cells are prepared from a suspension of parent cells. The plate with inserts is removed from the incubator. Using a wide bore pipette, the *Caco2Bbe1* cells (*n* = 25,000 per insert) are delivered in 0.5 mL of complete media to the apical side of each insert. Once the plate is full, a single slight shake is applied, causing newly plated cells to disperse evenly. These incubate for 7 days until the first of several feedings. The apical side’s media is replenished first, before adding fresh 0.5 mL media. Then, basal side’s media is replenished with fresh 0.5 mL media. In this way, the filters are prevented from drying during media changes. The plates are returned to the incubator and repeat feedings occur on days 9, 11, and 13. Each of these feedings is with complete media but on day 14 the apical filters are given 0.5 mL media containing 1% FBS (”deplete serum’’; Figures S1, S2 and S4–S6). Switching to deplete serum apically achieves a protein-enough concentration comparable to the lumen of the normal large intestine [16]. Deplete apical serum also helps keep the neurons—once added- from shriveling (a finding which varied depending on the numbers of *SH-SY5Y* cells plated; Figure S5). However, if FBS is removed completely from the apical media it leads to *Caco-2Bbe1′s* detachment from the inserts (personal observation and [16]). Note, however, that to this point the basal side remained in complete media (10% FBS, until later). The result was fully differentiated epithelium, operationally defined by having a high level of transepithelial electrical resistance (TEER). 

*What to watch for in this step?* This protocol largely follows a report by Delle and Dumas [29]. Consistent with that report, signs of *Caco2Bbe1* maturity begin as early as day 8. For instance, our inserts appeared fully confluent without cell division beyond day 8. Regarding the pH of the apical compartment, it was noted that the color of the media became golden just one day after refeeding from day 7 onward. This is believed indicative of epithelial cells secreting gastric acid apically. By day 9 it was noted that the apical volume dropped about 0.05 mL per day, corresponding to a rise in basal volume. This is indicative of polarized epithelial cells pumping water from top to bottom across the filter [30]. Also, we observed by day 11 most inserts had attained high transepithelial electrical resistance (TEER), indicative of tight junctions. TEER values continue to rise gradually to a plateau by day 13. Once TEER has plateaued in all the wells, the *Caco2Bbe1* cells are fully mature and ready for transplantation.

Step-2:  *SH-SY5Y* Plating

Come day-7 post-plating of the *Caco2Bbe1* inserts, a companion 24-well plate of *SH-SY5Y’s* needs plating. For most situations, this involves *n* = 400 *SH-SY5Y* cells/well. The procedure of plating is similar to the above. Once the wells are aliquoted, a single slight shake of the plate causes cells to uniformly disperse before going into the incubator. 

*What to watch for:* The more densely *SH-SY5Y’s* are plated, the more quickly stellate shapes and short neurites emerge. When plated at *n* = 400 cells/well and left untreated, 5 days is required to see these neuroblastoma-sized neurites. If plating *n* = 25,000 cells/well, the stellate shapes appear as quickly as 24 h of culture. This is because *SH-SY5Y* cells stimulate each other’s growth (though never achieving full-length neurites unless given RA). Noteworthy is that dividing *SH-SY5Y* cells do not need to retract their neurites when dividing. Instead, budding occurs from the somas of neurite-bearing precursors which detach and float to a new site. In an earlier study [26] we found the propensity of SH-SY5Y’s to bud new cells, as well to form neurospheres (another phenomenon of concern), varies with the brand, lot, and concentration of FBS, as well as with the stage of *SH-SY5Y* differentiation.

Step-3:  Making *SH-SY5Y* into neurons

From this step, the protocol closely follows the method of De Medeiros and colleagues [31]. After 5 days in monoculture, spent media was discarded. Without washing the cells, 0.5 mL of serum-free media was applied to contain 10 µM all-trans retinoic acid (RA; #MP021902696, MP Biomedical, Santa Ana, CA, USA). The RA stock (500× in 95% ethanol) is stable for 2 weeks at 4⁰C if kept away from heat, light, and air. After the addition of 10 µM RA, the cells were returned to the incubator for 2 days. This proved sufficient treatment, however additional supplementary treatments were tried experimentally. The additional supplementary treatments were 0.5 mL more serum-free FBS plus a second supply of 10 µM RA, or with human Brain-Derived Neurotrophic Factor (BDNF, NB27593910U, Fisher) dissolved at 50 ng/0.5 mL in the supplemental serum-free media with fresh RA. If photographing the cells, they should be returned to the incubator for at least 1 h before beginning Step–4. 

*What to watch for?* Full-length neurites become obvious throughout the wells by two days in RA. Using a second dose of serum-free RA has been advised by some [31] but it may not be needed according to our data. The reader is advised to review the time constraints we faced when adding BDNF (below and under Discussion). Also, neurites are fragile and, for this reason, as little mixing as possible is advised.

Step-4:  Co-culturing

This step begins when the two cell types are already fully differentiated. Namely, there should be a 24-well plate at hand filled with mature *Caco2Bbe1*-laden filter inserts that divide an apical compartment with fresh 0.5 mL depleted serum media from a basal compartment (at the moment with 0.5 mL complete media). The inserts should possess polarized epithelium with high TEER. At hand should also be a 24-well plate of 7-day-old *SH-SY5Y* neurons treated for 2 days with RA in open wells in 1 mL of serum-free media. Before proceeding, the volumes of the *SH-SY5Y* wells need be decreased by 0.5 mL to accommodate the incoming filters. The transplantation occurs by lifting with forceps each *Caco2Bbe1* insert out of its well and placing it gently into a corresponding *SH-SY5Y*-containing well. Left behind are epithelial droppings and spent media. Carried forward with the cells on the inserts is apical epithelial media. Bathing from below is the two-day spent RA serum-free media. Set in place, the RA containing serum-free media below begins to seep upward into the filter and mix with the media seeping downward with signals secreted by the epithelial cells. The neurons remain attached 1 mm below the epithelial cells on the bottoms of the wells. Four short plastic legs support the filter this distance. This sandwich configuration is returned to the incubator for an adaptation period of 15 h.

*What to watch for?* The long neurites pre-developed in the cultures should remain stable for 3 days after the first dose of RA, going forward with/without dose-2 of RA alone or RA ± BDNF, or with/without making the co-cultures. This allows a 3-day window of opportunity at the end of RA treatment-1 before any sign of neuron shriveling appears (more details about this under Treatments). A check of drop-through material under the *Caco2Bbe1* filters is also advised to ensure that no epithelial cells managed to get through the filters. The drop-through material will include cell fragments and debris. If actual *Caco2Bbe1* cells appear in the drop-through material, the inserts should be rejected.

Step-5:  Treatments

Depending on substance solubility and site of action in vivo, the model can be manipulated either apically or basally by adding to upper or lower chambers. If the experiment is to examine changes in neurite length as done herein, we recommend starting the *SH-SY5Y* neurites mid-length. This was achieved in the present study without having to pretreat with RA. The *SH-SY5Y* cells were left under *Caco2Bbe1′s* for 2 days which produced mid-length neurite outgrowth before adding the probiotics apically. 

### 2.7. Transepithelial Electrical Resistance (TEER)

TEER is a non-invasive electrical method for assessing the strength of tight junctions in confluent *Caco-2Bbe1* inserts [32]. We used a Millicell^®^ ERS-2 system (Fisher Scientific, Waltham, MA, USA). A cut-off > 180 Ω cm^2^ resistance was required because above this value, tight junctions exist [33]. No experiments were begun unless starting TEER values exceeded this cut-off. TEER measurements began 15 min after the *Caco-2Bbe1* inserts were taken from the incubator. TEER was recorded in duplicate and then the plates returned to the 5% CO_2_/37 °C incubator for at least one hour before the next steps. TEER was calculated by subtracting a blank insert and correcting for the surface area of the insert filters (0.6 cm^2^). 

### 2.8. Cell Counts

Cellometry^TM^ was performed to count and size the cells (Nexelcom Inc., Lawrence, MA, USA). The cells were washed in PBS and the wash volume saved in separate tubes (containing possible floating cells added later). The remaining attached cells were treated with trypsin-EDTA, 2 min or more, diluted, triturated, and pelleted. The resuspension was added back to the floaters to acquire a pool of all cell types (floaters + trypsin/triturated attachees). These underwent brief (5 min.) centrifugation at 500× *g*, room temperature (RT). These were resuspended in PBS, diluted 1:1 in trypan blue dye solution (Fisher), transferred to the 20 µL chamber of a hemocytometer slide, and counted in the cellometer (Nexelcom). The cellometer subtracted cellular debris and distinguished the percent live cells (trypan blue excluding) from dead cells (trypan blue internalizing). When the cell counts were too low (from 400–2000 *SH-SY5Y* cells per well), we manually counted the cells in each well by microscopy (10× magnification). 

### 2.9. Staining Caco2Bbe1

After experiments were done, the inserts were dried upside-down in a laminar flow hood. The next day they went through a sequence: fixation in methanol, staining with HEMA 3 solution-1 (Fisher CAT #122-911B: Eosin Y), and staining with HEMA 3 solution-2 (Fisher CAT #122-911C: Azure A and Methylene Blue). Unbound stain was removed by copious water washes. The filters were emptied and dried. They were examined by phase contrast microscopy. 

### 2.10. Staining with Crystal Violet

To assess numbers and dimensions of neurites, the wells were stained with crystal violet dye as described by Crowley and coworkers [34], gently so the neurites remained attached during pipetting. Once wells were empty of media (without a wash step), absolute methanol was added (1 mL, gently). This fixation lasted 20 min. at RT. Methanol was replaced with 1 mL/well of sterile water. This was removed and replaced with 1 mL of a crystal violet dye solution. Staining continued for 5 min at RT before the stain was removed and the wells washed thrice with sterile water. Stained plates were dried under the laminar hood and examined under the microscope.

### 2.11. Image Capture

Microscopy was performed with a Micromaster^®^ inverted phase contrast microscope (Fisher model 12-563-520) using a plate holder and mechanical stage fitted with a 10× eyepiece and 4×, 10×, 20×, and 40× objectives. Images were focused manually and saved through a camera using Micron USB 2.0 Beta Rev 1.09a software. The camera settings for unstained cells were as follows: exposure was manual 60.5; gain was manual 71; appearance was brightness 64.5/contrast, 100/saturation 52.3/hue 3.64; the white balance was red 50/green 29/blue 57. The images saved with Micron software included a calibrated mm ruler on the X and Y axes.

### 2.12. Image Quantification

Images at 40× magnification were manipulated with Micron USB2 software (Fisher) and converted to jpegs for analysis by ImageJ (NIH software version 1.38 (http://rsb.info.nih.gov/ij/ accessed on 28 September 2022)). Only cells with unambiguous borders were chosen for analysis. Because the *SH-SY5Y’s* tend to clump, this excluded a lot of cells. Nonetheless, at least 3 neurons that met criteria were traced from each image. Since at least 3 images from each well were analyzed, at least 9 criteria-meeting cells were traced per experiment per well. The shapes were traced using a QGeeM stylus pen (Queens, NY, USA) on an iPad (Apple, iOS 12.2 or above). This was accomplished by controlling the PC wirelessly with the Remote Desktop program (Google). The entire outline of each neuron was traced for area quantification, making note of how many neurites stemmed from the cell’s soma. The cell was then retraced just around the soma. Subtracting soma from total cell area, the neurite area per cell was calculated. Values were summed and averaged for each well, yielding average cell area, average soma area, average neurite area, average percent neurite area, and average number of neurites per cell in each well. Some jpegs were later converted to grayscale so that ImageJ could quantitatively assess stain intensity, but this appeared not to be a biological variable (personal observation).

### 2.13. Probiotic Treatments

Two lactobaccili strains were chosen based on reports of improvement in anxiety [35] and/or memory [36] in animal studies. These were purchased from ATCC: (1) *Lactobacillus rhamnosus* (Hansen) Collins et al. (ATCC^®^ 7469^™^), and (2) *Lactobacillus fermentum* Beijerinck (ATCC^®^ 9338^™^). These were individually grown in Lactobacilli MRS broth (BD 288130; ATCC) to prepare stocks, stored at −80 °C. Each probiotic was studied independently except in one combination experiment. A stock vial was thawed for 2 min in a 37 °C water bath, diluted 1:15 in MRS broth, and propagated overnight at 37 °C in an incubator without CO_2_ regulation. From this, a 1000-fold dilution was made in MRS broth. The viable bacteria were quantified in two ways: (1) immediate turbidity measurements of the cultures using a Nanodrop 2000/2000C V1.0 spectrophotometer (Thermo Fisher, Waltham, MA, USA); and (2) a series of dilutions smeared onto agar plates (Lactobacilli MRS Agar, BD288210; ATCC) so that after 2–3 days colonies could be counted. Based on growth curves constructed in this duplicitous manner, we foreknew the OD600 equivalence of a viable number of bacteria. Although the OD600 readings were quick approximations of plate counts, they allowed to add in the range of 10^7^ colony-forming units (cfu’s) bacteria to each well on the *C2BBe1* (apical) side of the model. 

## 3. Results

We first applied *SH-SY5Y’s* directly to *Caco2bbe1′s* grown to confluency and swiped in the middle of open wells of cells Figure S3). Swiping the *Caco2bbe1′s* beforehand had been done so that *SH-SY5Y’s* in the open zones would be readily observable from day to day; and a low density of neuroblastoma cells was added (*n* = 2000) so that 6 days later it was not difficult to count them under the microscope. Inevitably, however, the swipe zones differed in size from well to well—roughly taking up 25% of the space in each well. To adjust for this, we photographed the entirety of each well and measured the area in each swiped zone (Rasband, W.S., ImageJ, U. S. National Institutes of Health, Bethesda, MD, USA, https://imagej.nih.gov/ij/, accessed on 28 September 2022, 1997–2018). This allowed a determination of neuroblastoma numbers per unit area. A mathematical adjustment allowed to calculate the total numbers in each co-culture well. The numbers were found highly comparable to the control neuroblastoma cell numbers in companion open wells plated without *Caco2Bbe1′s*. This indicated that the neuroblastoma cells did not proliferate differently in direct co-cultures with complete media compared to monoculture. 

**Figure 1:**
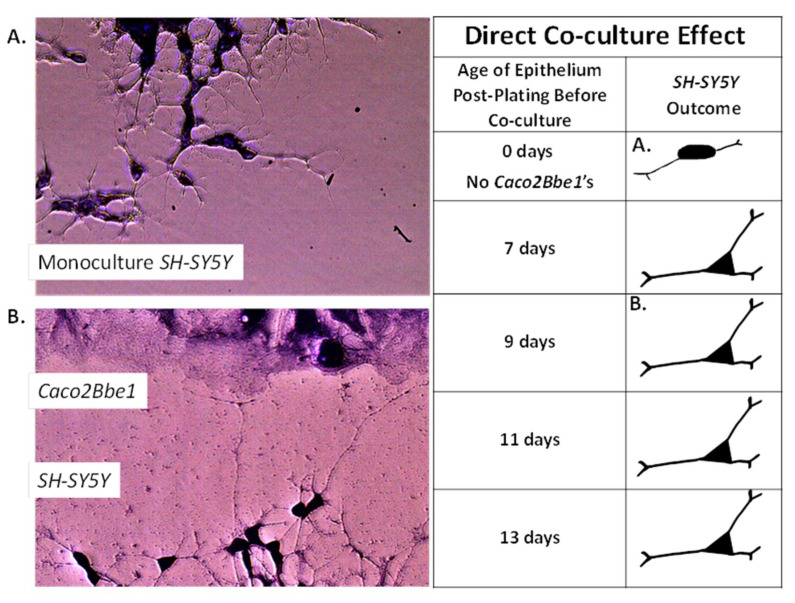
The *SH-SY5Y* neuronal phenotype in response to direct co-culture with *Caco2Bbe1*. The manner of swiping and assessing the *Caco2Bbe1* cells is described under Methods and in legends of Figure 2 and Figure S1. A suspension of *SH-SY5Y* cells was added to the swiped open wells of *Caco2Bbe1* cells (without inserts) in complete (10% FBS) media. The experimental variables were the presence and stage of epithelial cells prior to swiping. Six days after adding the *SH-SY5Y’s,* the media was removed by washing, the remaining cells were stained with crystal violet, and photographs were made using 40× phase contrast microscopy. The images show: (**A**) control *SH-SY5Y* cells in empty wells without *Caco2Bbe1′s* (same source, plating density, media, and time of growth), and (**B**) experimental *SH-SY5Y’s* cells with their neurites extending toward the *Caco2Bbe1* cells in co-culture. At the earliest epithelial stage of swiping (7-day-old *Caco2Bbe1*′s post-plating), the cells had reached confluency only one day prior. By comparison, based on studies performed later with transmembrane electrical resistance, the *Caco2Bbe1* cells should have achieved full maturity by 11 days in pre-culture. As shown in the drawings, all stages of *Caco2Bbe1* maturation were equally effective on neurite out-growth in the *SH-SY5Y* cell line.

**Figure 2:**
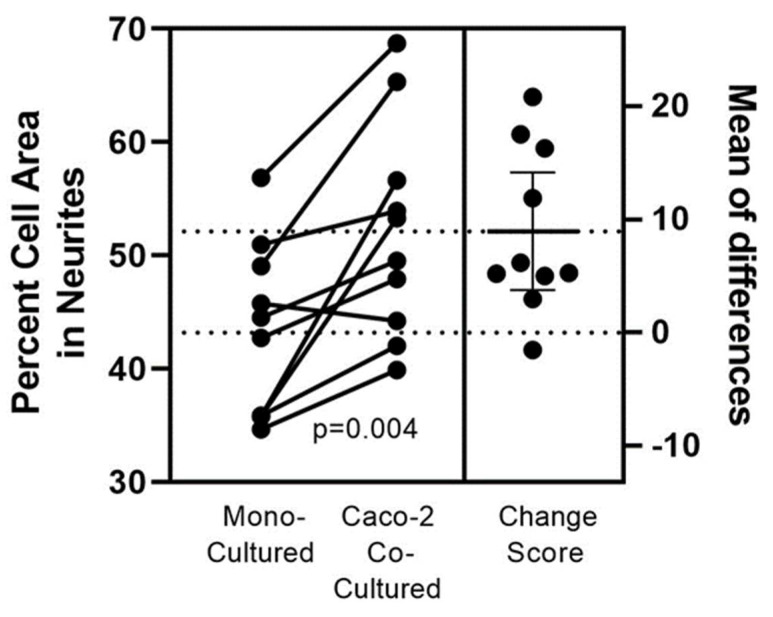
Quantifying the *Caco2Bbe1* effect on neurite out-growth in *SH-SY5Y* cells directly co-cultured. *Caco2Bbe1* (Caco-2) cells were plated at *n* = 200,000/well in 3 mL 6-well plates with complete 10% FBS media, incubated, and refed on day 6. On day 7 a single swipe was made in the middle of each well using flat end of a sterile p1000 pipette tip. Debris was removed and a fresh suspension of *n*= 2000 *SH-SY5Y* cells was added to each well along with fresh complete media (3 mL per well). Paired controls were set-up with *SH-SY5Y* cells in monocultures. Following 6 more days as co-cultures, media was removed, and the attached cells were stained with crystal violet. More details of this set-up may be found in Figure S1. Photographs were made at 40× magnification and *SH-SY5Y* cells were traced using ImageJ software. At least 5 neurons in each swiped area were chosen for tracing based on finding enough neurons that did not clump or overlap with other cells. The left Y-axis is the neurite percent of total cell volume. The number of neurites per cell was also tabulated but not shown (described in the text). Each dot represents average values from 3 wells per experimental condition (so at least 15 neurons were drawn per dot). Connected pairs of dots correspond to paired groups in each experiment. The *p*-value derives from a paired two-tailed *t*-test using all (*n* = 10) experiments. The right Y-axis shows mean percent differences between each pair.

Figure 1 displays co-cultured *SH-SY5Y* cells along the epithelium swipe line. The *SH-SY5Y* cells were well-tolerated and not diminished when co-cultured in pre-swiped *Caco2Bbe1* wells. Indeed, their neurites appeared attracted to the epithelial cells and cells were shaped slightly differently than monocultured. The neuroblastoma phenotype in co-culture (Figure 1B) possessed longer neurites and smaller somas compared to controls (Figure 1A). The numerical basis for this finding is shown in Figure 2. These direct co-cultures showed 10% more neurite percent of *SH-SY5Y* area regardless which day the *Caco2Bbe1* cells had been swiped (days 7, 9, 11, or 13 after their plating shown in Figure 2). Furthermore, the *Caco2Bbe1* cells had barely grown into the swipe zones even without co-cultured *SH-SY5Y’s*. This later finding matches a previous study [28], meaning that once the *Caco2Bbe1* cells achieve confluence on the filters, they almost completely stop dividing.

**Figure 3:**
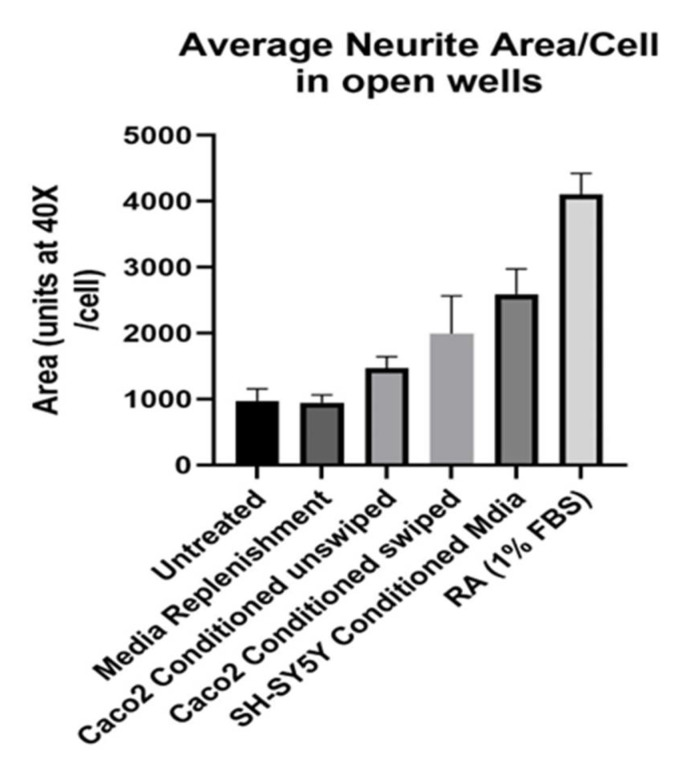
Conditioned media promotes longer *SH-SY5Y* neurites. *SH-SY5Y’s* were plated in open 24-well plates at low density (*n* = 400/well) in complete (10% FBS) media. The experimental variable was type of media change on day 6. Namely, from left to right, the wells were either: untreated (no media change or addition); replenished with fresh complete media; replenished with complete conditioned media taken from confluent *Caco2Bbe1* cells (not swiped); replenished with complete conditioned media taken from confluent *Caco2Bbe1* cells swiped 2 days earlier; replenished with complete conditioned media from near- confluent *SH-SY5Y* cells (not swiped); or subject to switching to a media low (1%) in FBS containing 10 µM retinoic acid (RA). Two days afterward, media was removed, cells were washed and stained with crystal violet, and photographs were made using 40× phase contrast microscopy. Cells were traced and the average neurite area per cell was computed using ImageJ software. Bars are means ± SEMs. Significant *p* values by two-tailed, unpaired head-to-head *t*-tests were as follows: Media Replenishment alone vs. *Caco2Bbe1* conditioned media unswiped (*p* = 0.019). Media replenishment alone vs. *Caco2Bbe1* conditioned media swiped (*p* = 0.0004). Media replenishment alone vs. *SH-SY5Y* conditioned media (*p* < 0.0001). Media Replenishment alone vs. RA in 1% FBS (*p* < 0.0001). There were no significant differences between *Caco2Bbe1* conditioned swiped vs. unswiped (0.091).

Attention turned next to conditioned media. The swiping studies had been informative, but some possibility remained that the neurites had arisen from swipe trauma rather than normal cell-to-cell signaling. Moreover, the co-culture design of Figure 1 and Figure 2 started with a suspension of *SH-SY5Ys*, making neurites the result of a complex process starting from floating cells. Hence, we sought to determine if conditioned media from *Caco2-Bbe1* cells could also work to lengthen neurites of attached neuroblastoma cells. As shown in Figure 3, both scraped and unscraped conditioned media proved equally efficacious (controls with media replenishment alone vs. *Caco2bbe1* conditioned unswiped (*p* = 0.02); but not significantly different from swiped vs. unswiped, *p* = 0.09). As well, a finding came from homologous conditioned media from *SH-SY5Y* confluent cells. The homologous conditioned media proved more stimulatory on neurite outgrowth than *Caco2bbe1* conditioned media (*p* < 0.0001 to media replenishment alone). Also, the efficacy of RA was first studied at this stage of our project (effective on neurites; *p* < 0.0001 compared to media replenishment alone). In summary, unswiped conditioned media worked well to induce neurites. This implied that any trauma due to the swipe itself was insufficient to explain the results of Figure 1 and Figure 2. It is noteworthy that these studies were conducted in complete media using an immature phenotype of *SH-SY5Y* without the need for treatment with RA.

**Figure 4:**
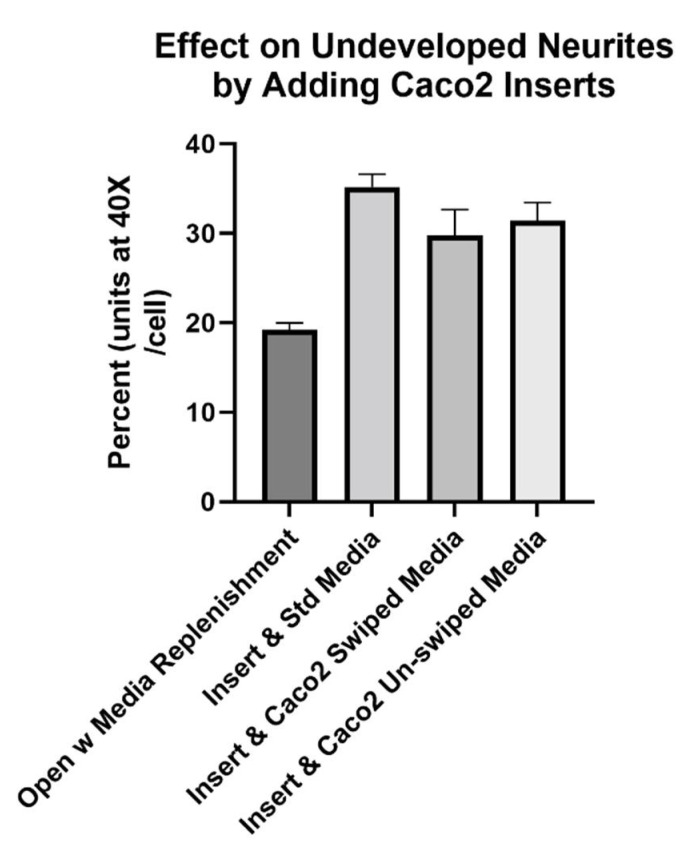
*Caco2Bbe1* inserts, alone, induce higher percent of neurites in *SH-SY5Y* cells. *SH-SY5Y’s* were plated in open wells at low density (*n* = 400/well) in complete (10% FBS) media. They received inserts on day 6. The experimental variable that occurred on day 6 was insert addition and/or the type of media under the inserts. Namely, from left to right, either: remained open wells but replenished with standard complete (10% FBS) media; added *Caco2Bbe1* inserts at the same time as replenished basally with standard complete media; added *Caco2Bbe1* inserts at the same time as replenished basally with complete conditioned media taken from confluent *Caco2Bbe1* swiped 2 days earlier, or; added *Caco2Bbe1* inserts at the same time as replenished basally with complete conditioned media taken from confluent *Caco2Bbe1* cells which had not been swiped. Two days afterward, the media was removed, cells were washed and stained with crystal violet, and photographs were made using 40× phase contrast microscopy. Cells were traced and the average neurite area percent was computed using ImageJ software. Bars are means ± SEMs. All three experimental bars are significantly higher than media-only controls (*p* < 0.01). The right three bars were not significantly different from each other.

At the beginning of studies with *Caco2Bbe1*-laden filter inserts, we set-up the model a bit short of the final protocol. That is, we did the steps excepting the *SH-SY5Y’s* in serum-free media and inducing with RA. The result proved once again that neurites grow longer when exposed to *Caco2Bbe1.* In this case, the neurites were longer on *SH-SY5Y* cells 2 days after administration of *Caco2Bbe1* inserts compared to without inserts (Figure 4). An additional finding was lack of enhancement by adding conditioned media (swiped or unswiped) with the *Caco2Bbe1* inserts (Figure 4). 

**Figure 5:**
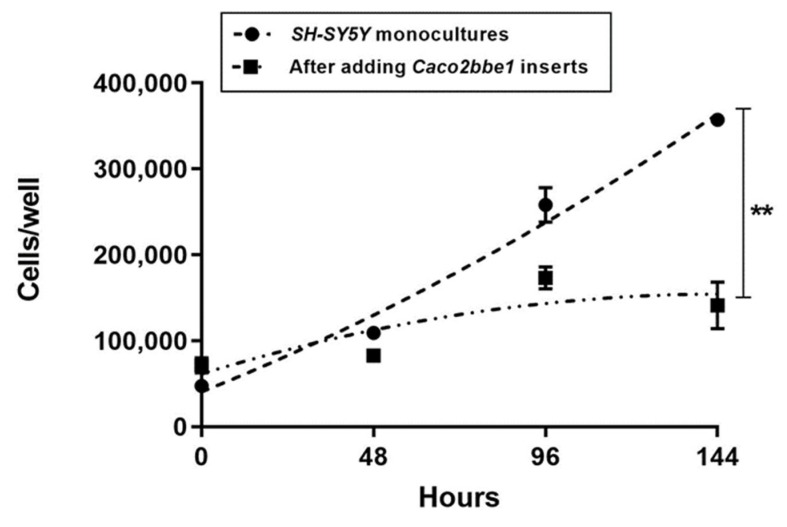
Cytostatic effect of *Caco2bbe1* inserts on growth of *SH-SY5Y* cells. *Caco2BBe1* cells were plated on inserts (*n* = 25,000/insert) and grown with regular feeding of complete (10% FBS) media, top, and bottom. On day 11, *SH-SY5Y* cells were plated in separate open 24-well plates (*n* = 25,000/well) in complete media. Three days later, both 24-well plates were refed complete media. Immediately afterward, experimental wells received *Caco2Bbe1* inserts. The control wells of *SH-SY5Y* cells remained without inserts (monocultures). Three or more wells per the condition of *SH-SY5Y* cells were harvested and counted by trypan blue cellometry at 0 (day of combination), 48, 96, and 144 h. Plots are means ± SEMs. The asterisks indicate high statistical difference in cell numbers between the two groups at 144 h (*p* < 0.001).

Having successfully documented neurite enhancement by *Caco2Bbe1* on *SH-SY5Y*, we wondered if this was the only modification on the neuroblastoma cells? To answer this question, we counted cells and looked closely at where the cells were in relation to each other before and after transplanting inserts. We prepared *Caco2Bbe1* inserts identically except the *SH-SY5Y’s* were plated at a higher density (*n* = 25,000 per well compared to *n* = 400 cells/ well in previous studies). We also bypassed Step 3 (RA treatment). We performed transplantations with 11-day-old inserts atop 2-day-old *SH-SY5Y* cultures, keeping complete media under the inserts. Cellometry was performed on the lower wells after 48, 96, and 144 h. Surprisingly, the results revealed cytostasis in the *SH-SY5Y* cells in response to *Caco2Bbe1* inserts (Figure 5). However, cytostasis only occurred when the cells were in complete media. Additionally, we found that the neuroblastoma cells in complete media responded to C*aco2Bbe1* inserts by de-clumping and spreading (Figures in Supplementary Figures S7–S9). This too was contingent on the presence of complete media. When complete media was replaced with serum-free media, cystostatic and spreading effects did not occur. This way, the neurite-promoting effects of *Caco2Bbe1* inserts were seen in isolation of these other media-dependent changes. 

**Figure 6:**
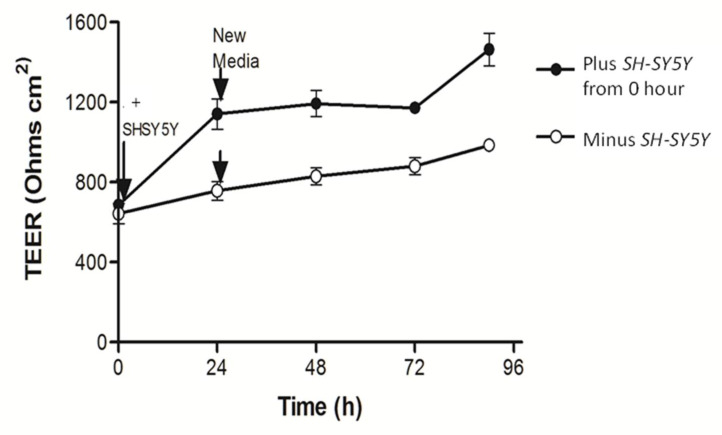
*SH-SY5Y* cells underneath cause tighter junctions between *Caco2bbe1* cells on inserts. *Caco2BBe1* cells were plated on inserts (*n* = 25,000/insert) and grown with regular feeding of complete (10% FBS) media, top and bottom. On day 11, *SH-SY5Y* cells were plated at high density in separate open 24-well plates (*n* = 25,000/well) in complete media. Four days later, both 24-well plates were refed complete media and half the well inserts were transplanted above the *SH-SY5Y* cells. The experimental variable was presence or (continued) absence of *SH-SY5Y* cells under the inserts. The measure was daily TEER readings. Three inserts were assessed per condition and the experiment was repeated three times. Evidence that the initial rise in TEER did not owe to the simple replenishment of media is shown by the arrows when fresh complete media was again replaced after 24 h. No elevation in TEER appeared by just replenishing the media. The inserts not transplanted above SH-SY5Y cells never caught up in TEER. Plots are means ± SEMs.

We next explored if anything happens in reverse. Could the *SH-SY5Y* neuroblastoma cells exert a bottom-up effect on *Caco2Bbe1* epithelial cells? The manner in which we tested this is depicted in Supplementary Figure S6. We started with standard *Caco2Bbe1*-laden inserts and separately plated *SH-SY5Y’s* at *n* = 25,000 per well. We again held-off adding RA in 1% FBS. The transplantations were done with 13-day-old inserts atop 4-day-old *SH-SY5Y* cultures, keeping complete media under the inserts. TEER, which started high, was assessed daily for 4 days. The results shown in Figure 6 indicate that the presence of *SH-SY5Y* cells rapidly raised *Caco2Bbe1* TEER about 33% over 24 h. TEER remained at this level for at least 2 more days. Control *CacoBbe1* inserts without *SH-SY5Ys* never caught up. This was unrelated to the use of fresh media before transplantation because when more fresh media was added after an additional 24 h, no TEER changes happened (Figure 6).

**Figure 7:**
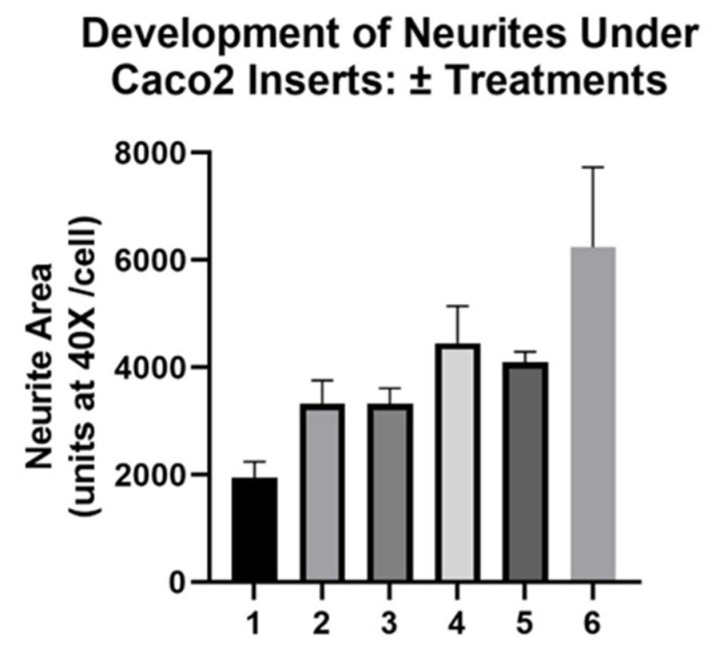
Development of long neurites under *Caco2Bbe1* inserts in response to retinoic acid. *Caco2BBe1* cells were plated on inserts (*n* = 25,000/insert) and grown with regular feeding of complete (10% FBS) media, top and bottom, sufficient to attain high TEER. Eleven days after their plating, *SH-SY5Y* cells (*n* = 400) were plated in a different 24-well plate in complete media without inserts. Fourteen days after *Caco2Bbe1* plating, the apical side of the inserts was switched to media with 1% FBS, and most of the *SH-SY5Y* open wells were replaced with media lacking FBS (0%). On the same day-14, the open wells of *SH-SY5Y* without FBS received 10 µM retinoic acid (RA). Two days later (day 16), the first two conditions were assessed for neurites: (1) untreated open wells in complete (10% FBS) media, and (2) open wells switched to media lacking FBS but containing RA. Among the remaining wells, some were given a second dose of RA and simultaneously covered with *Caco2Bbe1* inserts in apical 1% FBS to be assessed another 2 days later (on day 18). Other wells received a combination second treatment with 10 µM RA + 50 ng/0.5 mL Brain-derived Neurotrophic Factor (BDNF) to be assessed three days later (6 below). Before reaching that stage, the day-18 data were collected from cells given (3) 2 × 2-day basal treatments with RA with the second treatment going under the inserts in 1% FBS, and (4) 2 × 2-day basal treatments with RA with the second going under the inserts reverted to 10% FBS. After this, (5) another day passed and the readings from the inserts in 1% FBS were reassessed (on day 19). (6) Assessments were at last made of the wells that had been treated first open for 2 days with RA alone and then simultaneously with inserts and basal RA+ BDNF for 3 more days after that (until day 19). In each case, the *SH-SY5Y* cells were washed and stained with crystal violet and photographed using 40× phase contrast microscopy. Cells were traced and average neurite areas were computed using ImageJ software. Bars are means ± SEMs. All experimental bars (2–6) are significantly higher than media-only controls (*p* < 0.01). The right five bars were not significantly different from each other.

**Figure 8:**
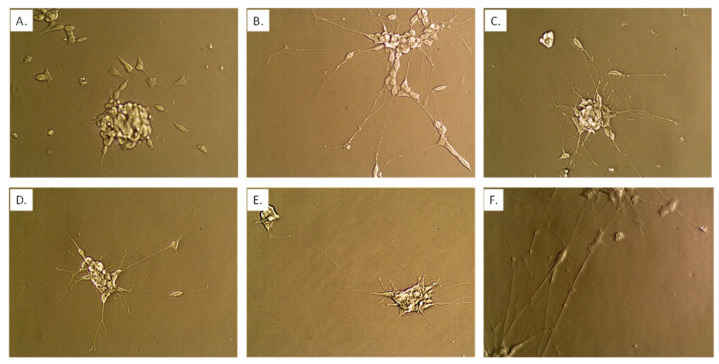
Neuronal state using co-culture model begun by plating n = 25,000 *Caco2Bbe1* cells on inserts in each well of a 24-well plate. Such were regularly refed to attain high TEER as described in Figure S2. Eleven days after starting to grow the inserts, a separate 24-well plate was given *SH-SY5Y* cells (*n* = 400) in complete (10% FBS) media. On day 14 after plating the inserts, the apical media was switched to deplete media with 1% FBS (basal media remained at 10% FBS). On the same day, media in the *SH-SY5Y* wells was replaced with fresh media lacking FBS (0%) containing 10 µM retinoic acid (RA). Some controls remained in complete media. Both plates were returned to the incubator for 2 more days. At this time, the untreated panel (**A**) and 2-day RA-treated panel (**B**) neuroblastoma cells were photographed at 40× magnification. A second batch of serum-deplete media + RA was applied to some of the experimental *SH-SY5Y* cells (becoming 0.5 mL volume) and inserts were transplanted atop them; although there were 2 more variations (no second dose of RA but adding inserts, or a second dose of RA but remaining in open wells). After two more days in the incubator, the inserts were briefly lifted so that neurons could be photographed without staining at 40× magnification. Panel (**C**) represents the effects of a single dose of RA then two more days under inserts. After two more days, the inserts were again briefly lifted so neurons could again be photographed panel (**D**). After two more days, the inserts were again briefly lifted so neurons could again be photographed panel (**E**). The image in panel (**F**) comes after two scheduled doses of RA without inserts placed atop them and then incubated the same time course as panel (**E**). The set-up recommended for treatment studies is probiotics begun with panel (**C**) (state of neurons when probiotics were administered) and ending with panel (**D**) (control neuron state compared to the end of studies).

As intriguing as these interactions are between *Caco2Bbe1* and *SH-SY5Y*, even more clarity came when inserting Method Step-3, which allowed neurite actions to be viewed independently. Method Step 3 may seem to focus on RA. Indeed, the addition of RA promptly converted the *SH-SY5Y* cells into mature neurons with full-length neurites and compact soma (Figure 7 and Figure 8). But RA itself, was not the key to revealing neurite extensions *independently* of confounds. It was the removal of serum. When the *SH-SY5Y* cells were placed in serum-free media as requisite for RA to act, the absence of serum simultaneously halted cell division. It was the ongoing ability of cells to divide that had previously caused the cytostatic and cell spreading findings which were confounding our understanding the neurites (Figure 6). Without serum in media, we realized that neither of these confounding phenomena occured in response to the *Caco2bbe1′s*. [Two asides: (1) the absence of cell division also became essential when we added the probiotics; and (2) we later experimented with a higher FBS level of 11% in apical media only to discover that the rise in serum led to an exaggeration of the previous finding of top-down promotion by epithelium for longer neurites in *SH-SY5Y* (similar Figure 7, compare bars 3 and 4)]. Put another way, once the serum was removed we became confident that any neurite enhancements would not be confounded when treating (i.e., with probiotics) to secondary cytostasis and spreading-out responses due to the epithelium.

We explored a second dose of RA for 2 more days with or without adding BDNF These extra steps marginally enhanced the lengths of the neurites beyond the first 2-day treatment with RA alone (Figure 7 bar 6; Figure 8F). For practical reasons we preferred a simpler model completed 2 days and 15 h after a single dose of RA in serum-free media. The last 15 h of this time was used for adapting the newly transplanted cell lines to each other and to the deplete serum. Once fully set-up, the model remained stable with the same length neurites for 56 h (Figure 8).

**Figure 9:**
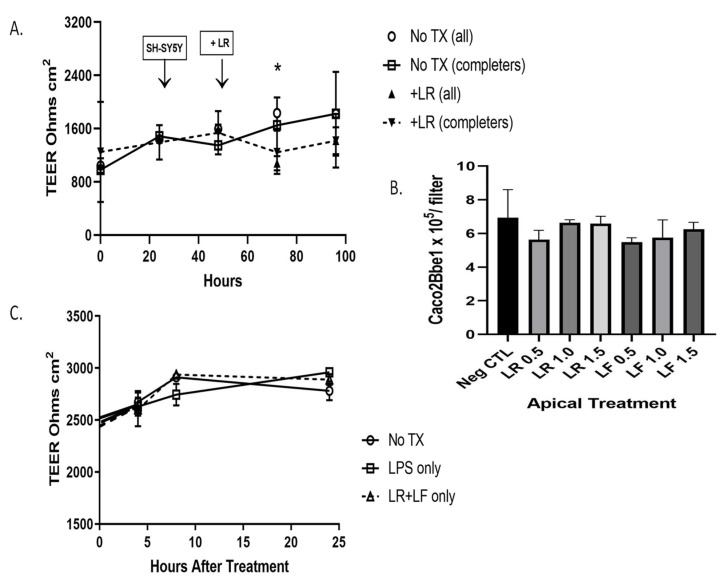
Status of *Caco2Bbe1* cells during probiotic administration to the apical side of co-culture model. These studies began by plating *n* = 25,000 *Caco2Bbe1* cells on inserts in a 24-well plate. Such were regularly refed (Figure S2). Eleven days after starting the inserts, a separate open-well plate was given *SH-SY5Y* cells (*n* = 400) in complete (10% FBS) media. On day 14 after plating the inserts, the apical media was switched to media with 1% FBS (though basal media remained 10% FBS). On same day, the media in the open *SH-SY5Y* wells was replaced with fresh media lacking FBS (0%) but containing 10 µM retinoic acid (RA). Both plates were returned to the incubator for 1 more day. Panel (**A**) shows TEER values from one day after open-well RA. The experimental *Caco2Bbe1* inserts were transplanted atop *SH-SY5Y* cells (first arrow). A day after that, probiotic *Lactobacillus rhamnosus* (LR) was added atop the inserts, or, in the untreated case, no LR was added (just vehicle: 1% FBS media). Some of the starting inserts were harvested and counted before reaching the 190-h endpoint, which meant the data analysis was performed from start to finish in two ways: with “all” or “completer” wells. Panel (**B**) shows *Caco2Bbe1* cell numbers counted from the inserts harvested at 190 h. The groups were either without probiotics, or with LR added at 0.5, 1.0, or 1.5 × 10^7^ bacilli per insert, or with *Lactobacillus fermentum* (LF) added at 0.5, 1.0, or 1.5 × 10^7^ bacilli per insert. Panel (**C**) shows another experiment in which TEER was followed for 24 h from when the probiotic was added (set up as in panel (**A**)), except the experimental variables were without more additions, or with only lipopolysaccharides (LPS) added apically (0.5 µg in 0.5 mL per well), or with a mixture of LR and LF added apically, each at 0.5 × 10^7^ bacilli per 0.5 mL volume into the upper well of the insert (No LPS). The plots are means ± SEMs. * Comparing TEER in panel (**A**), with LR versus without LR (all), *p* = 0.033 when adjusted for multiple unpaired t tests. No other comparisons were statistically significant in any of the panels.

Before beginning studies with probiotics we sought to ensure that no detrimental effects occur at the level of epithelium-laden inserts. We set up the model as before and apically applied either or both of two probiotic bacteria strains, *Lactobacillus rhamnosus* (LR) or *Lactobacillus fermentum* (LF). These were applied in the range of 10^7^ colony-forming units (cfu’s) per strain per insert. Panel A of Figure 9 shows that the addition of LR did not alter TEER for 48 h studied. Panel B of Figure 9 shows that over a range of doses of LR or LF, there were no effects on the numbers of *Caco2Bbe1* cells remaining on the inserts. Panel C of Figure 9 shows two additional aspects. One is that the combined apical treatment of LR and LF was unable to alter TEER. The other finding is that adding lipopolysaccharides (LPS) was unable to alter TEER. LPS is not produced by lactobacilli but we thought to test it because it is an inflammatory toxin released by toxic bacteria. Beyond this, we also stained the *Caco2Bbe1* filters following treatments to determine if the probiotics had elicited more subtle cellular changes. The inserts were stained with H&E stain. We discerned no obvious effects of the probiotics at this level of staining (Figure S10). 

**Figure 10:**
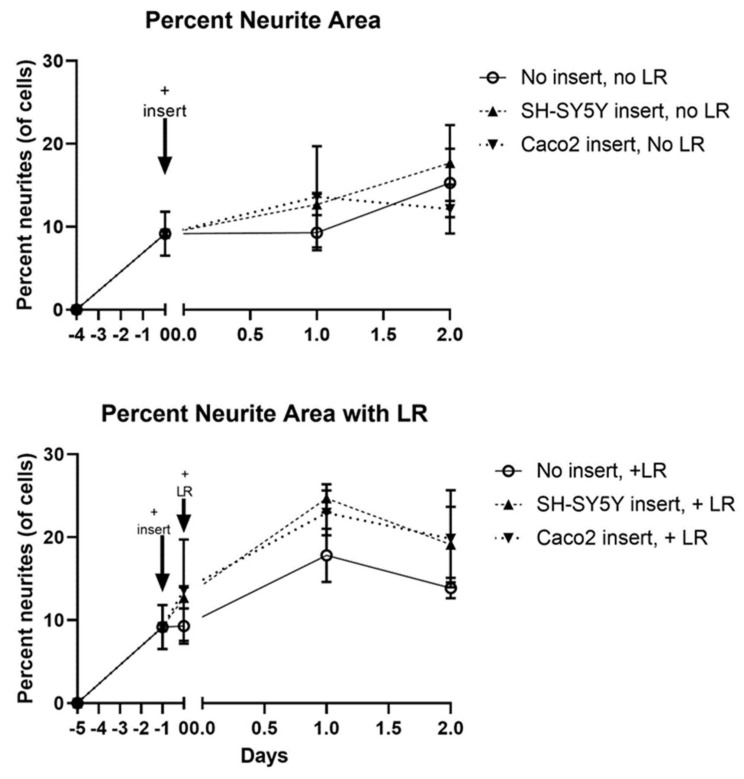
Time course transmembrane effects of *Lactobacillius rhamsosus* (LR) on *SH-SY5Y* neurites. Inserts were set up according to the plating and feeding sequence (Figure S4), whether using normal *Caco2Bbe1* cells (*n* = 25,000) or control *SH-SY5Y* cells (*n*= 25,000 inserts). After 9 days (designated “minus 5 days” on the lower graph), companion *SH-SY5Y* wells were plated (*n* = 50/ well) into open wells of a separate 24-well plate with complete (10% FBS) media. Three days later, the apical media of all inserts was changed to 1% FBS. The next day (day -1 on the lower graph), the first 40× microscopic photographs were taken of the *SH-SY5Y* cells, following which the inserts were added to create the 3-dimensional (3D) co-cultures. It should be noted that this experiment intentionally used neuroblastoma cells with medium-length neurites because had *SH-SY5Y’s* been converted to their full-length neurite phenotype before adding LR, it would have been impossible to observe a rise in neurite length as seen in the lower panel. Upper panel: Transmembrane control of apical 1% FBS on basal neurites. The data follow the percent of neurites per total cell volume according to type of inserts with apical 1% FBS in the media, either in: 13-day-old blank inserts, 13-day-old *SH-SY5Y* inserts, or 13-day-old *Caco2Bbe1* inserts. Lower panel: LR transmembrane effect on basal neurites. Fifteen hours after the model became 3D (still running in parallel with upper panel), LR was applied apically (*n* = 1.0 × 10^7^) to all inserts. The time course followed the percent of neurites per total cell volume according to type of inserts, starting as either: LR on 13.6-day-old blank inserts, LR on 13.6-day-old *SH-SY5Y* inserts, or LR on 13.6-day-old *Caco2Bbe1* inserts. Plots are means ± SEMs. Across all conditions and time points investigated, *Caco2Bbe1* TEER values remained highly indicative of tight junctions (not so with blank or *SH-SY5Y* inserts that failed at any time to register TEER; data not shown here but fully expected since *SH-SY5Y* cells do not form tight junctions).

**Figure 11:**
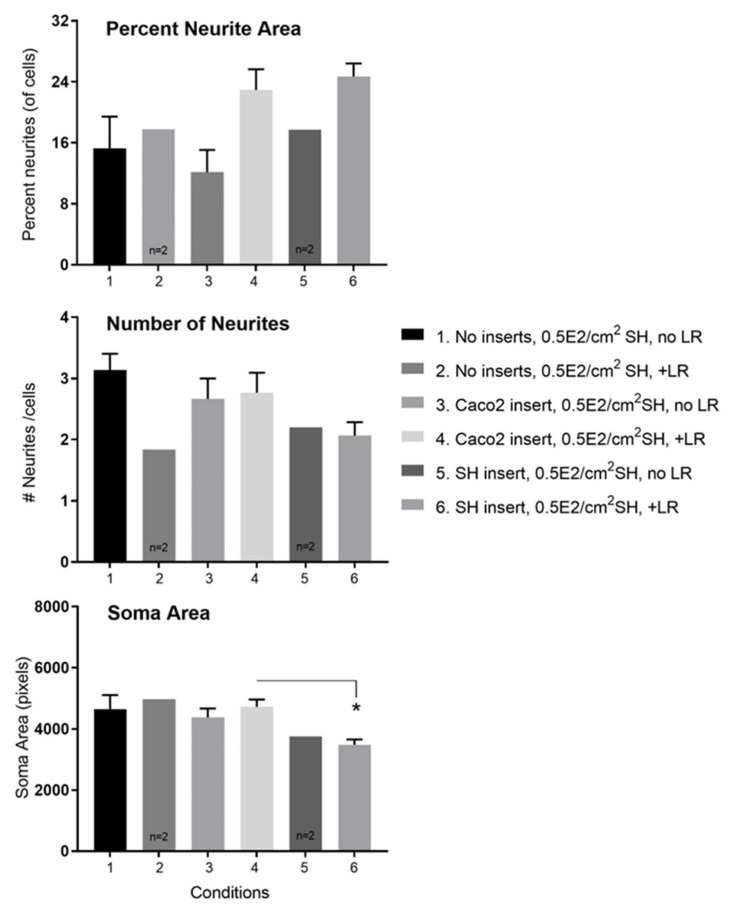
Details of changes induced by transmembrane *Lactobacillus rhamnosus* (LR) on *SH-SY5Y* neurons in tri-cultures. The data derive from the same experiment set-up in Figure 10 at 24 h post-LR. Upper panel: The apical addition of LR caused a rise in percent neurite area per total cell volume with either *Caco2Bbe1* or *SH-SY5Y* inserts compared to inserts without LR, or to blank inserts with or without LR. Mid panel: The apical addition of LR did not cause a rise in number of neurites per cell with any inserts. Lower panel: The apical addition of LR to *Caco2Bbe1* insets did not change soma volume per neuron. However, the apical addition of LR to *SH-SY5Y* inserts lowered soma volume per neuron in wells under the *SH-SY5Y* inserts. # is used to abbreviate numbers (of neurites). Bars are means ± SEMs. * Comparing soma areas under conditions 4 versus 6, *p* = 0.031 by Tukey’s multiple comparisons test. Regarding overall ANOVA, treatment between columns yielded *p* = 0.149. Although this slice of time effect alone did not meet criteria for significance, the pattern of higher neurite area in conditions 4 and 6 is a static validation of Figure 10 time course.

The final step of our model—adding probiotics apically where they can transmit signals to neurons—required careful consideration due to tri-culturing *Caco2Bbe1*, *SH-SY5Y*, and probiotics. To distinguish these studies from what was done earlier, we will refer to the tri-cultures as 3-dimensional cultures (3D). We set up a control group of the filter inserts laden with *SH-SY5Y* cells. We, of course, retained the main experimental condition with *Caco2Bbe1* inserts above neurons, that showed high enough TEER before adding probiotics. [The *SH-SY5Y* insert control group did not attain TEER since neurons do not form tight junctions]. We added the probiotics apically and examined neurites basally over time with and without inserts, or with and without probiotic. The results of these 3D studies are shown in Figure 10 and Figure 11. When comparing the upper panel to the lower panel in Figure 10, it is clear that the probiotic led to a higher percentage of neurites after 24 h. This rise in neurites was independent of whether the inserts contained *Caco2bbe1* cells or *SH-SY5Y* cells (Figure 10). In no case was there evidence that the lactobacilli passed across the filters. The rise in neurites caused by the lactobacilli was still clear but not so statistically significant after 48 h with the apical probiotic (Figure 10).

Figure 11 provides more insight into the type of neurite enhancement due to apical LR. From quantifying neurites, we determined that apically added LR leads to higher neurite areas per neuron (upper panel Figure 11). However, there was no increase in number of neurites per neuron (mid panel of Figure 11). Nor did the presence of LR on *Caco2Bbe1* inserts lead to a change in soma area of the neurons (lower panel of Figure 11). A drop did occur though in soma area of the neurons when *SH-SY5Y* inserts were given apical LR (also shown in the lower panel of Figure 11). Taken together, the results indicate that the transepithelial effect of LR is to increase the area of neurites rather than increase the number of neurites per cell. In other data, cellometry revealed that neither LR nor LF caused changes in cell numbers, mean cell diameters, or percent viabilities in either the *Caco2Bbe1’s* on the inserts or the *SH-SY5Y’s* in the lower wells (data not shown).

## 4. Discussion

We present a model of microbial signaling to enteric neurons as likely happens in the human large intestine. It is important to realize that the filter inserts possessed a pore size (0.4 micron) that restricted cells from migrating from one side of the unit to the other. Only molecules up to microvesicles might be possible candidates to traverse the filters. Plus, the *Caco2Bbe1* line grown atop the filters had tight junctions, assessed in our hands by high TEER, such that even lactobacilli were not able to penetrate from upper to lower chamber. We may conclude that any neuron changes resulting from additions made apically must have passed through the epithelial cells (transepithelial signaling). 

*Caco2bbe1* cells reach confluency in 6 days and continue to mature without need of inducing agents, until attaining high TEER by day 11 in monoculture on inserts [37]. For the sake of a stable plateau of TEER, we refed the cells until day 14 when the apical chamber was also given depleted (1%) serum. This was done for several reasons, but mainly to better model the gut lumen. After that, the inserts were transplanted atop pre-prepared monocultures of *SH-SY5Y* cells. Pre-prepared means that for 48 h prior the *SH-SY5Y* cells in separate wells had been treated with 10 µM RA in serum-free media. They showed elongated neurites of a distinctly different appearance than the neuroblastoma phenotype. With inserts atop them, the neurons were residing on the bottom of wells a mere 1 mm below *Caco2Bbe1* epithelium. The co-cultures were equilibrated 15 h to ensure the tight junctions remained, before the probiotic, or any sort of, treatment [27]. Left untreated, long neurites were stable for 56 h. Even after 24 h of apical treatment with LPS their lengths were not diminished (nor was the epithelium’s integrity compromised). For studies of the induction capacity of neurites, we started with intermediate neurite lengths. This was so that data of probiotics could observe plus or minus neurite changes (Figure 10 and Figure 11).

Readers trained to add antibiotics to mammalian cell cultures may well wonder if the addition of LR or LF led to cell demise and/or collapse of the 3D model. In fact, the results tended in the opposite direction. That is, epithelial TEER values rose high and neurites grew longer basally after the probiotics were added. There is precedence on TEER effects in previous reports [38,39,40]. The probiotics were thus well-tolerated in tri-cultures with *Caco2bbe1* and *SH-SY5Y* cells.

The main endpoint for most of our paper was neurite length. Our main finding was neurite lengthening in response to probiotic lactobacilli applied apically to the model (Figure 10 and Figure 11). As a positive control, neurite lengths were shown to increase appropriately in monocultures treated with RA. Since longer neurites are considered to be better, our results are in keeping with prevailing views about probiotics leading to a healthy gut [41]. Of course, the results are just model results. It remains to be determined whether similar lengthening occurs at nerve endings of the GBA when luminal probiotics begin to signal upward to the brain [42,43].

A better understanding of the GBA is needed in hope of developing new treatments for diseases of the GI tract [44]. There is already evidence that the GBA is involved in autism spectrum disorder, amyotrophic lateral sclerosis, transmissible spongiform encephalopathies, Parkinson disease, Alzheimer’s disease, and major depression [2]. There is even evidence that the gut microbiome explains aspects of human temperament and personality [7]. Having a good model of microbiome-to-GBA signaling may connect the dots between the microbiome and these disorders [15,16]. Someone might question if our model is as cholinergic as the literature says it should be when formed from RA treatment alone [31,45]. Although the fact was not rigorously proven in the present paper, we obtained preliminary data with a choline esterase inhibitor, chlorpyrifos, indicating that our *SH-SY5Y’s* were indeed cholinergic. Apoptotic-like cell death was seen within 24 h in all the *SH-SY5Y* wells under inserts in a dose-responsive manner using 25–100 µM chlorpyrifos (unpublished). By contrast, the *Caco2Bbe1* cells on the inserts remained normal even in 100 µM chlorpyrifos even after 3 days (also unpublished). This agrees with the literature on chlorpyrifos that the agent kills only cell types that secrete acetylcholine and have cholinergic receptors.

Sepsis is a vexing condition not only because of overwhelming infection, but because a late-stage lethality often arises even after the bacteria are destroyed by antibiotics. Late-stage victims appear locked into a destructive pro-inflammatory spiral that kills them [45]. Related to this is one of the gut’s main arginine metabolites, made in copious amounts by toxic bacteria, known as agmatine [46,47]. In a 2001 animal study of sepsis, a derivative of agmatine (agmatine-aldehyde) was given pharmacologically to model septic animals and found to save the animals [48]. Agmatine is a nicotinic cholinergic antagonist and anti-inflammatory molecule often found at high levels in the GI tract [49]. So, could the therapeutic value of this compound in the 2001 animal study of sepsis be due to unblocking a cholinergic pathway of the GBA [9]? Although a link between the cholinergic anti-inflammatory pathway and sepsis has been known for about 20 years [9], it has been difficult to study because anti-cholinergic therapies have side effects. We believe our new model system could be used to test the hypothesis that agmatine could treat sepsis through acting on the GBA. Namely, in a modified version of our model, with toxic bacteria added apically, one could screen molecules that are agmatine enhancers to treat the late-stage inflammatory process of a model of this disease [47].

Homage certainly needs be paid to the many studies upon which our ideas are based. It was for instance known long before our work began that the Caco-2 subclone, *Caco2Bbe1*, holds advantage because of its uniform cell shape and lower mucus production compared to the parent Caco-2 line [50]. It was known that *Caco2Bbe1* cells grown for 11 days on inserts reliably convert into a monolayer of polarized intestinal enterocytes [29]. These had been shown to organize with tight junctions, microvilli, and pumping transporters [29,51]. The stability of *Caco2Bbe1* inserts was also well-established, shown by retaining tight junction resistance even when co-cultured with basal-side immunocytes or apical-side probiotics [52]. Our study is beholden to all these prior findings. Moreover, monocultures of *SH-SY5Y* cells have long been known to produce neurites under certain conditions [27]. The best-known inducers are phorbol esters, RA, or BDNF [53,54]. One previous study examined monocultured *SH-SY5Y’s* treated with RA, showing differentiation into a neuroblast state (cholinergic in nature) which could be even more fully differentiated (dopaminergic in nature) after adding BDNF along with RA [55].

What kind of signals might explain our results? Many neurotransmitters and modulators are known to exist luminally in the gut [22,56]. A range of cytokines is known to be emitted by epithelial cells, macrophages, and neurons in the gut [14]. A list of candidate dietary molecules of importance would also have to include food tryptophan metabolites [57], membrane precursors and cofactors [58], and caffeine [59]. Regarding the gut microbiome, it is also known that bacterial wall peptidoglycans are released as direct signals to the ENS [60]. There are also short-chain fatty acids and bile salts thought active in lumen-to-nerve signaling [61,62]. There are so many signaling pathways to explain the findings of our model [15].

Previous studies with *SH-SY5Y* cells have mostly pertained to the CNS, particularly to dopaminergic neurons involved in Parkinson’s disease [63]. Those studies often used BDNF. By comparison, we focused on RA-only treatments because RA given alone is known to cause neuroblastoma cells to differentiate into cholinergic neurons [31]. Of course, cholinergic nerves are also important in the CNS, but few dopaminergic neurons exist in the GI tract [56,64]. 

The importance of depleted serum (1% FBS) apically and serum-free media basally has been emphasized throughout this paper. However, serum-free media may not be universally required for neurite-inducing agents to act on *SH-SY5Y* cells [31]. In fact, our direct co-culture studies (Figure 1, Figure 2, Figure 3 and Figure 4) were performed in complete (10% FBS) media, and they showed neurite inductions (Figure 1, Figure 2, Figure 3 and Figure 4). It was just that those studies were confounded by concomitant cessation of cell division and de-clumping. As well, although the apical inserts were in deplete (1%) FBS, our experiments with LR and LF (Figure 10 and Figure 11) were intentionally performed in basal complete (10% FBS) media. This was done to create a starting phenotype of mid-length neurites, capable of changing in either direction. Phorbol esters (i.e., TPA) are also known to act on *SH-SY5Y’s* in the presence of 20% FBS to induce long neurites following 2 × 48-h doses (personal observations, [54]. It is possible TPA might allow a better ‘workaround’ for the main limitations of our model. That being the case, the removal of serum in our model was very instructive because it created a study of non-dividing cells wherein neurite changes could not be misconstrued as secondary to cell growth changes. The sum of it is that depending on the application, the experimenter may opt to use complete media with 10% FBS.

## 5. Conclusions

A first-of-kind in vitro model is described for studying transepithelial gut lumen-to-nerve signaling as likely occurs in the intestines. We believe this an important step towards discovering how the microbiome signals across gut epithelium, eventually upward via the GBA to the brain. The model displays face validity on several levels. Firstly, the epithelial model cells, themselves, were found to induce neurite outgrowth from short to mid-sizes. Secondly, we document the ability of probiotics to transepithelially induce neurite lengthening from mid-size to longer-size in the neuron model cells, which fits current understandings about probiotics. In addition, the neurons are likely to be cholinergic based on previous literature and our own (unpublished) work. The model is easy to set up, its tools are widely available, and we hope it will be applicable to a variety of research questions.

## Supplementary Materials

The following supporting information can be downloaded at: https://www.mdpi.com/article/10.3390/nu14224856/s1. Figure S1: Unsuccessful attempt of conditioned media to enhance the top-down promotion of neurites by epithelial inserts; Figure S2: Final steps to optimize the co-culture system; Figure S3: Effect on neuroblastoma cells after 6 days of direct co-culture with model gut epithelial cells in open wells; Figure S4: Neuroblastoma phenotype after 3–6 days of co-culture with epithelial inserts varied by apical media; Figure S5: Value of low FBS on *Caco2Bbe1* inserts prior to co-culturing above near-confluent *SH-SY5Y* cells; Figure S6: Bottom-Up effects from *SH-SY5Y* in combination with basal media on *Caco2Bbe1* TEER; Figure S7: Plate view of the anti-neuroblastoma model when maturated *Caco2Bbe1* inserts co-culture above near-confluent *SH-SY5Y* cells in 10% FBS media; Figure S8: Microscopic view of anti-neuroblastoma model when maturated *Caco2Bbe1* inserts are co-cultured above near-confluent *SH-SY5Y’s* remaining in 10% FBS media; Figure S9: ImageJ results of alternative anti-neuroblastoma model when maturated *Caco2Bbe1* inserts are co-cultured above near-confluent *SH-SY5Y* cells remaining in 10% FBS media; Figure S10: Microscopic comparison of mature *Caco2Bbe1* cells on day-21 in the fully developed model.
