# Supplementary material for: Transepithelial Effect of Probiotics in a Novel Model of Gut Lumen to Nerve Signaling
Source: Nutrients. 2022 Nov 17;14(22):4856. doi: 10.3390/nu14224856 (PMC9697698; doi:10.3390/nu14224856)
Supplement: Supplementary file 1 [file nutrients-14-04856-s001.zip › nutrients-1969660-supplementary.pdf]

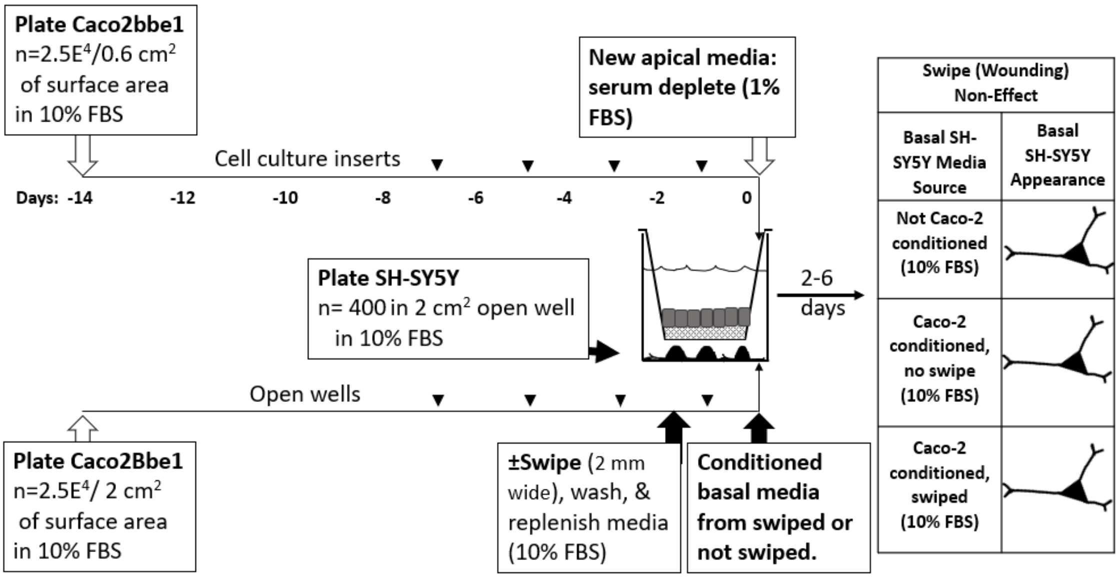

Figure S1. Unsuccessful attempt of conditioned media to enhance the top-down promotion of neurites by epithelial inserts. Shown is the neuroblastoma phenotype during co-culture with epithelial cell inserts under different preparations of basal media.. Experiments began in routine manner by plating: (1) n=25,000 *Caco2Bbe1* cells on inserts in each well of a 24-well plate, and (2) n=25,000 *Caco2Bbe1* cells in the open wells of a different 24-well plate. On day-12, each well in the open 24-well *Caco2Bbe1* plate was swiped, washed, and replenished with complete media. On day-14, the apical media of all inserts was switched to 1% FBS and the inserts were transplanted atop a third 24-well plate *of SH-SY5Y* cells that had been plated 4 days earlier at n= 400 cells per well without inserts; plated in complete (10% FBS) media but switched just prior to the inserts according to experimental variables. The variables were types of basal media: either normal complete media (10% FBS), conditioned media from wells of unswiped *Caco2Bbe1* cells (10% FBS), or conditioned media taken from wells of swiped *Caco2Bbe1* cells (10% FBS). *SH-SY5Y* controls which underwent all but a last basal media replenishment step, looked the same (Supplementary Fig S4). Inverted triangles in the figure point to refeeding with complete media (10% FBS). Following 2-6 days co-culture, the attached cells were photographed, counted, and neurite areas traced for volume determinations as described under Methods. **Results:** The hypothesis was that switching the media of *SH-SY5Y's* to a *Caco2BBe1* conditioned media would push the cells toward the full neuronal phenotype. This was based on the observation that exposure to *Caco2Bbe1* had, alone, moved the *SH-SY5Y* phenotype in the neuronal direction (Supplementary Figure S3). However, the *SH-SY5Y's* ended no more (or less) neuron-like compared to the normal insert controls. This non-effect of adding swiped conditioned media tends to rule out any concern that the neurites seen in Suppementary Figure S3 owed to the swipe process itself.

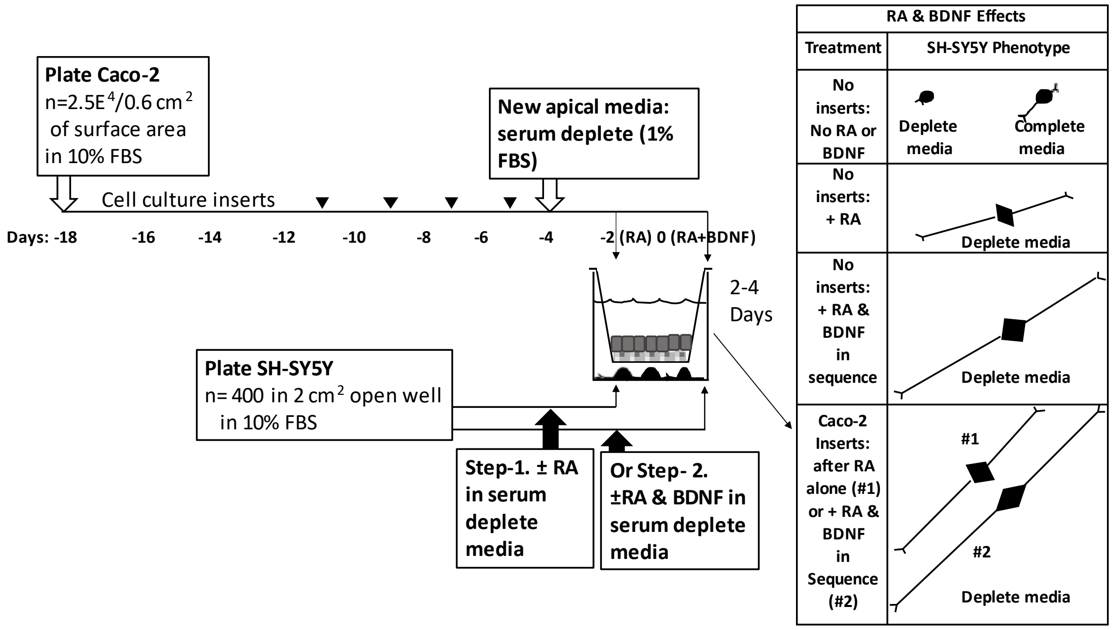

Figure S2. Final steps to optimize the co-culture system. This flowchart differs from Supplemental Figure S1 by now pretreating the *SH-SY5Y* cells in basal media depleted of FBS with the neurite-inducing agent retinoic acid (RA) alone or in combination sequence with Brain-Derived Neurotrophic Factor (BDNF), before adding the inserts. The flowchart differs from Figures S3, S5 and S6 because the number of neuroblastoma cells is lower. This experiment began by plating n=25,000 *Caco2Bbe1* cells on inserts in each well of a 24-well plate. Eleven days later, *SH-SY5Y* cells (n=400) were plated in a second 24-well in complete media without inserts. On day-14, changes were made to the media of both plates. Namely, the apical inserts were switched to media with 1% FBS and the *SH-SY5Y* media was replaced with media lacking FBS (0%), though it contained 10 μM RA. On day-16, two experimental variations occurred: (1) Some inserts were transplanted atop neuroblastoma cells which had a fresh batch of basal serum-deplete media with or without RA, or (2) some inserts remained alone to await treatment-2 of the remaining open wells of maturing *SH-SY5Y* cells given either a fresh batch of media lacking FBS (0%) with or without supplement of 10 μM RA + 50 ng/0.5 BDNF. These 'waiting' inserts were transplanted two days later atop the other last open-well neurons. The inverted triangles in the figure point to refeeding with complete media (10% FBS). At the end, attached cells were photographed, counted, and neurites traced for area determinations as described under Methods. **Results:** The longest neurites were seen when pretreating *SH-SY5Y* cells for 2 days in open wells with RA followed by RA+BDNF for 2 more days and then adding the inserts (cell #2). However, this set-up offered limited utility because by the time the inserts were added the *SH-SY5Y* cells had existed in serum-deplete media so long that just one day later the long neurites started to detach from the plate. Moreover, neurites made this way were not so much longer than neurites made using RA alone (either after one dose for 2 days or after a second dose with RA given with the inserts at the same time). In the case of cell #1, the long neurites were induced by RA for only 2 days, and this allowed another 2+ days to experiment before the depletion of FBS would lead to neurites detaching from the dish (personal observation). .

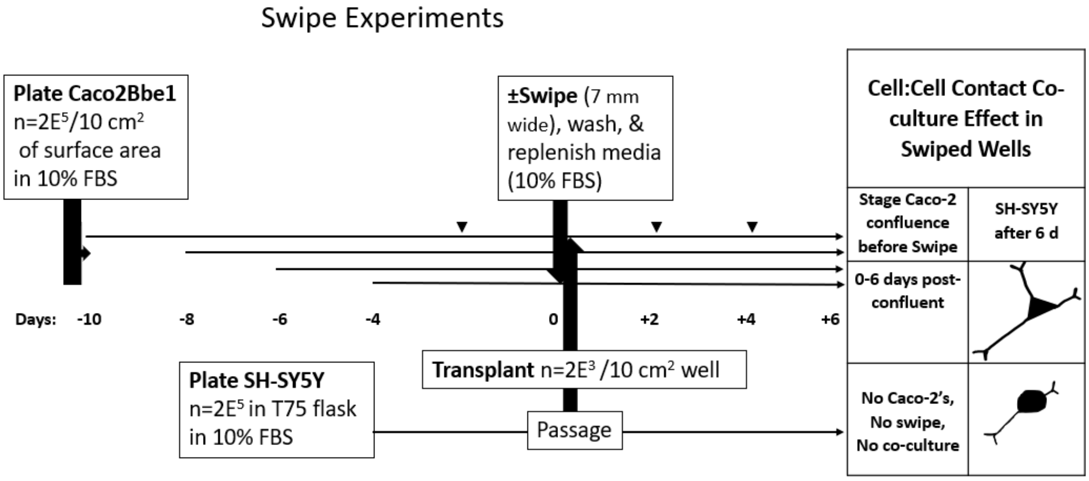

Figure S3. Effect on neuroblastoma cells after 6 days of direct co-culture with model gut epithelial cells in open wells. The first step was plating n = 200,000 *Caco2Bbe1* cells in each well of 6-well plates with complete 10% FBS media. The experimental variable was length of time *Caco2Bbe1's* grew before swiping and adding *SH-SY5Y* cells: either 4, 6, 8 or 10 days post-plated *Caco2Bbe1's*. Control *Caco2Bbe1's* were swiped but did not receive the neuroblastoma suspension or they received a cell suspension with n = 2,000 *Caco2Bbe1's* instead (homologous treatment control). A single swipe was made in the middle of each well using the flat end of a sterile p1000 pipette tip. Debris was removed by repeated washes and the outline of the swipe was indelibly traced on the bottom of each well. Shortly thereafter a freshly made suspension of n= 2,000 *SH-SY5Y* cells was added to experimental wells from a 4-day flask in 10% FBS media. The same *SH-SY5Y* cells were plated in empty wells as controls. The inverted triangles point to days when refeeding occurred with complete media (10% FBS), to ensure that the *Caco2Bbe1* cells and *SH-SY5Y* cells grew well. Following a 6-day co-culture period, the attached cells were photographed, counted, and neurite areas traced for area determinations as described under Methods. The overall area of each swipe was also measured by ImageJ software. The distance the *Caco2Bbe1* cells had grown from the swipe lines was recorded in three places per well. **Results:** The presence of *Caco2Bbe1* cells tended to transform *SH-SY5Y* cells toward a neuronal phenotype, although short of the long neurites seen in studies with retinoic acid added. Compared with the *SH-SY5Y* monoculture controls, the neuroblastoma cells in the swipe zone were more neuron-like: i.e., more neurites per cell and more neurite area per cell (results in main paper). There was no obvious difference in neuroblastoma cells in relation to length of time the *Caco2Bbe1* cells had been grown before swiping. The *Caco2bbe1* cells did not grow far from their swipe line.

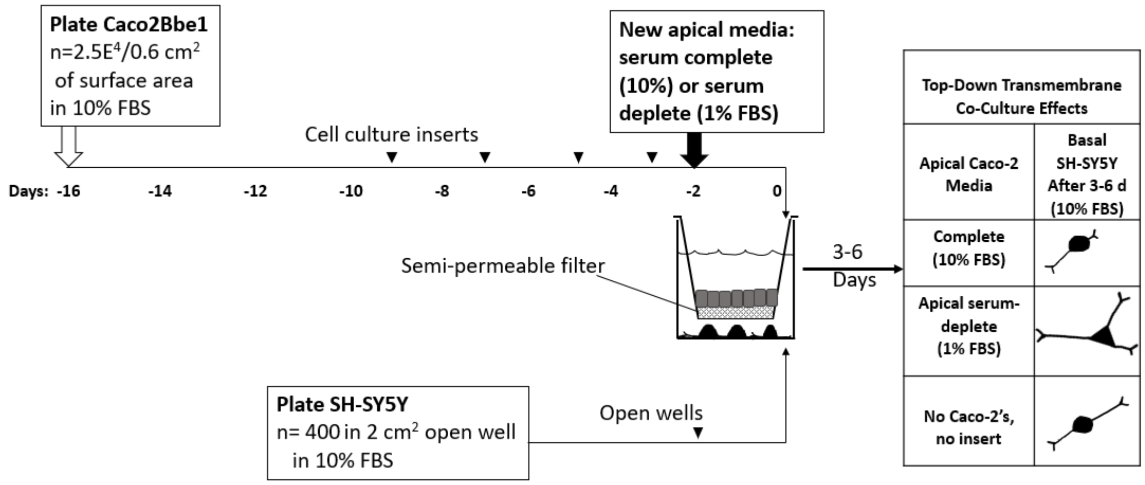

Figure S4. Neuroblastoma phenotype after 3-6 days of co-culture with epithelial inserts varied by apical media. The graphic depicts two procedural variations which informed toward the ultimate neuronal co-culture model (Figure S2). Step-1 was plating *n* = 25,000 *Caco2Bbe1* cells on inserts in each well of 24-well plates. The experimental variable was replacing apical complete media with 1% FBS on day-14, versus refeeding the inserts with complete (10% FBS) media on day-14. On day-16 the inserts were transplanted atop *SH-SY5Y* cells that had been plated 6 days earlier at *n* = 400 cells per well of 24-well plates in complete (10% FBS) media without inserts. Some *SH-SY5Y* cells also continued in empty wells as controls. The inverted triangles of the figure point to refeeding with complete media (10% FBS). Following 3-6 days of co-culture, the attached cells were photographed, counted, and neurite areas were traced for volume determinations as described under Methods. **Results:** When complete (10% FBS) media was held constant, the neuroblastoma cells under the inserts appeared shriveled compared to *SH-SY5Y* monoculture controls. But, switching the apical media 2 days prior to 1% FBS, led neuroblastoma cells under the inserts to appear more neuron-like compared to the comparison *SH-SY5Y* conditions.

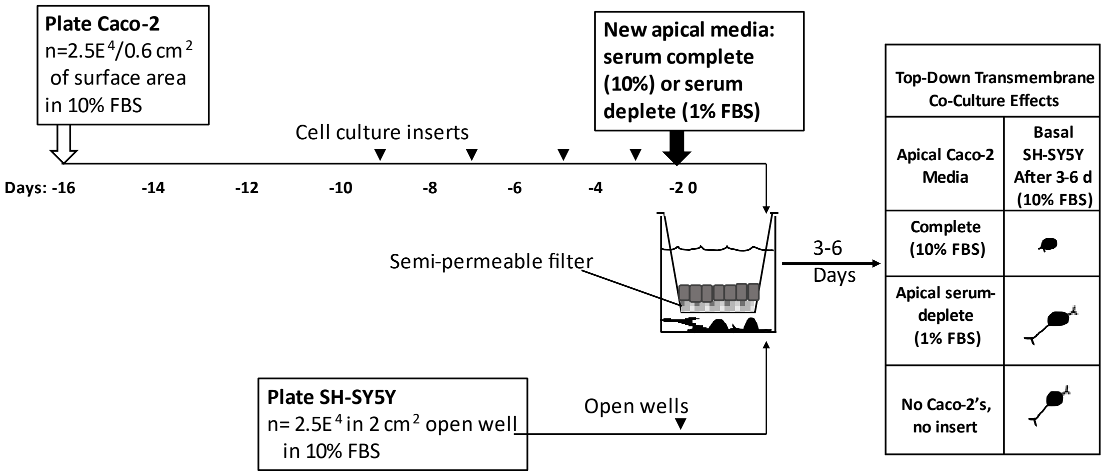

Figure S5. Value of low FBS on *Caco2Bbe1* inserts prior to co-culturing above near-confluent *SH-SY5Y* cells. This graphic depicts a variation of Figure S4 using high numbers of *SH-SY5Y's*. The role of switching to 1% FBS in apical media is displayed even while two other factors – known keys to the preferred neuronal model in Figure S2 – were not optimized. Namely, this experiment used *SH-SY5Y* cells that were 62.5 X more densely plated than the preferred neuronal model and retinoic acid was not added to induce the outgrowth of long neurites prior to adding *Caco2bbe1* inserts. The control condition is with *Caco2Bbe1* inserts in 10% FBS media. The inverted black triangles point to refeeding times with complete media which allowed *Caco2Bbe1* cells to attain high TEER which remained high even after apically existing in 1% FBS media for 2 days on day 16. **Results:** The switch to lower (1%) FBS in the apical media of *Caco2Bbe1* cells did not alone lead to neurite outgrowth in underlying neuroblastoma cells. The benefit of 1% FBS apical media was seen firstly in preventing *SH-SY5Y's* from shriveling some 3-6 days after the *Caco2Bbe1* inserts were added – shown when the inserts remained in complete 10% FBS serum media. The reason for shriveling when co-cultured with *Caco2Bbe1* in apical 10% FBS remains unclear, especially since the swipe experiments showed no such phenomenon (Supplementary Figure S1), however this insert-selective phenomenon occurs in concert with other things; *SH-SY5Y* cytostasis (Figure 5 of main paper), a rise in TEER induced by enough *SH-SY5Y* cells (Figure 6 of main paper), and several other changes shown in the Supplementary Figures S7 - S9. We could describe the low (1%) FBS as anti-neuroblastoma. But the switch to low (1%) FBS was for two other reasons: (1) to more closely mimic protein concentration in the intestinal lumen and (2) to make possible to detect neurite changes in the absence of confounding changes in cell growth and spreading (Figures S7-S9).

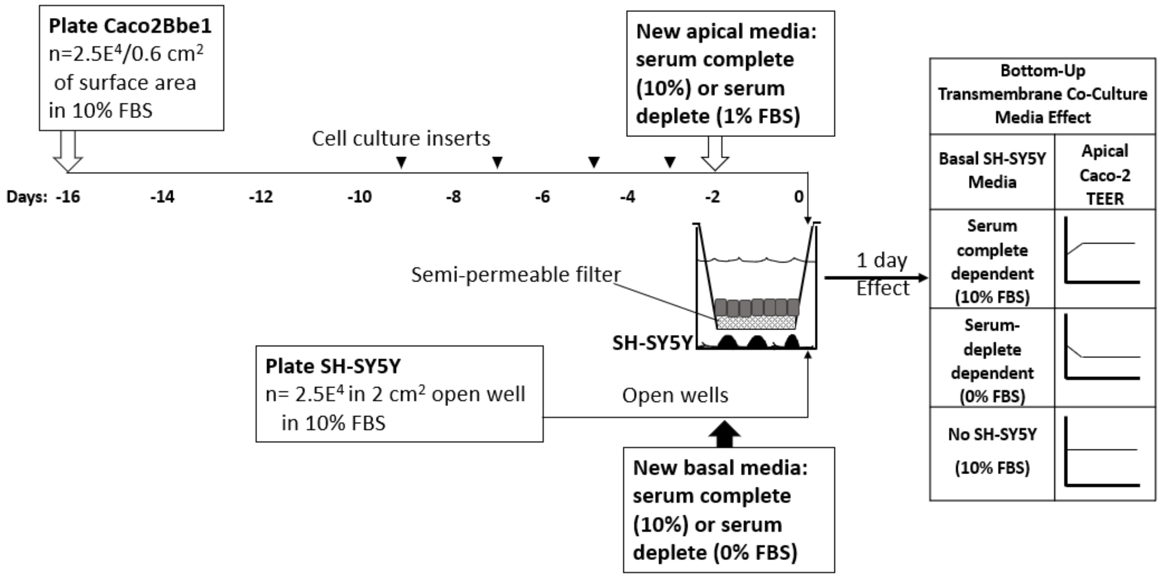

Figure S6. Bottom-up effects from *SH-SY5Y* in combination with basal media on *Caco2Bbe1* TEER. The flowchart differs from Supplementary Figures S1, S2 and S4 by using 62.5X more *SH-SY5Y* cells in the co-cultures. Experiments began by plating n=25,000 *Caco2Bbe1* cells on inserts in each well of a 24-well plate. Ten days later, *SH-SY5Y* cells (n=25,000) were plated into a second 24-well in complete media without inserts. On day-14, changes were made to the media of both plates. Namely, the apical inserts were either replenished with complete (10% FBS) media or switched to media with 1% FBS, while the *SH-SY5Y* cells were either replenished with complete (10% FBS) media or switched to media with 0% FBS. Two days later, baseline TEER readings were made. The inserts were then transplanted atop the neuroblastoma cells. TEER readings continued every 24 hours. Upside down triangles are days of refeeding with complete media to attain mature *Caco2Bbe1* high TEER. **Results:** Top panel: 24 hours after creating co-cultures with high numbers of *SH-SY5Y's* in 10% FBS (both apical and basal), TEER values were 20% higher, which maintained for 4 days. This effect was dependent on having high numbers of *SH-SY5Y's* since no TEER changes occurred when co-cultured with n= 400 *SH-SY5Y* cells (data not shown). Mid panel: 24 hours after creating co-cultures with 10% FBS apically and 0% FBS basally, TEER values were found 30% lower, which maintained for 4 days. This appeared to be independent of high numbers of *SH-SY5Y's* since it also emerged with n= 400 plated *SH-SY5Y* cells. Note that these panels only show TEER when maintaining 10% FBS in the apical compartment. When the apical media was switched from 10% FBS to 1% FBS while maintaining 10% basal FBS, a comparable result was obtained to that with serum-deplete basal media. Also note, if apical media went to 1% FBS and basal media went to 0% FBS, TEER values with high numbers of *SH-SY5Y's* after 24 hours were no longer indicative of tight junctions and did not recover. Yet, this was not found in our optimal model at n= 400 *SH-SY5Y* cells, because of the fewer cells. In that case, TEER dropped 50% lower to a new steady state over 48 hours since 0% FBS was imposed, but remained level and high enough to still be indicative of tight junctions. Overall, the results revealed that with high numbers of *SH-SY5Y* present, signals emerged from below that affected TEER values.

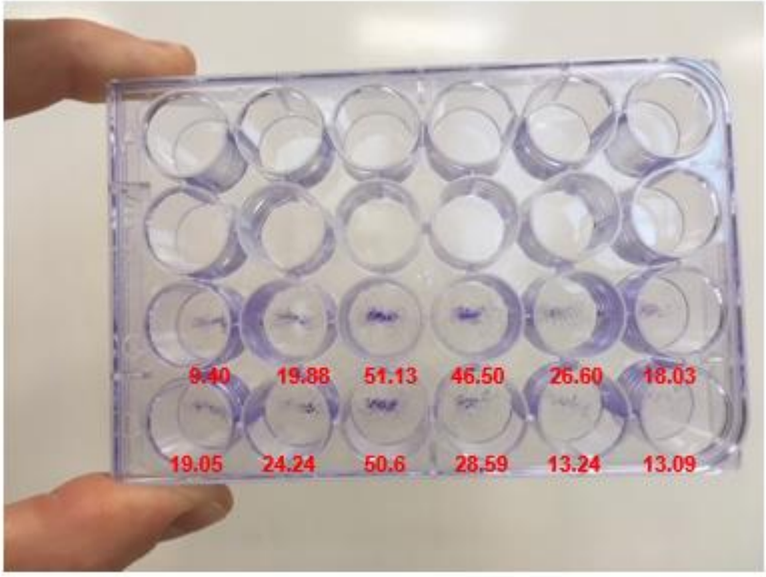

Figure S7. Plate view of the anti-neuroblastoma model when maturated *Caco2Bbe1* inserts are co-cultured above near-confluent *SH-SY5Y* cells in 10% FBS media. The co-culture set-up in wells C3, C4, D3, and D4 was identical to Figure S5 using the complete 10% FBS option. The main distinctions are that the *SH-SY5Y* cells were plated 62.5 X more densely compared to Figure S2 and they were encouraged by the presence of complete media to remain dividing when the inserts were added. The controls were: (1) *SH-SY5Y* cells without added inserts (wells C1 and D1), (2) *SH-SY5Y* cells with cell-free inserts with complete media apically (wells C2 and D2), and (3) *SH-SY5Y* cells with *SH-SY5Y*-laden inserts (wells C5, C6, D5, and D6). At the outset, when the inserts were added (on day-4 post *SH-SY5Y* plating), there existed the classic neuroblastoma phenotype with short neurites and lots of clumping. The inserts were set above the *SH-SY5Y* cells for only 24 hours. Immediately after removing the inserts, the attached cells were stained with crystal violet. Because a 24-hour exposure to *Caco2Bbe1* inserts should be insufficient to cause cytostasis (main paper Figure. 5), we expected no changes. Yet, more staining was evident to the eye in line with the presence of *Caco2Bbe1* inserts, confirmed by ImageJ analysis (red ink optical density numbers). Only after microscopic inspection did this paradox resolve itself, owing to neuroblastoma cells de-clumping & spreading due to *Caco2Bbe1* inserts. This effect led to cells covering more surface area than before the co-cultures were made, and this caused the wells to take more stain. Note that the cells were too numerous to count or assess neurites per cell.

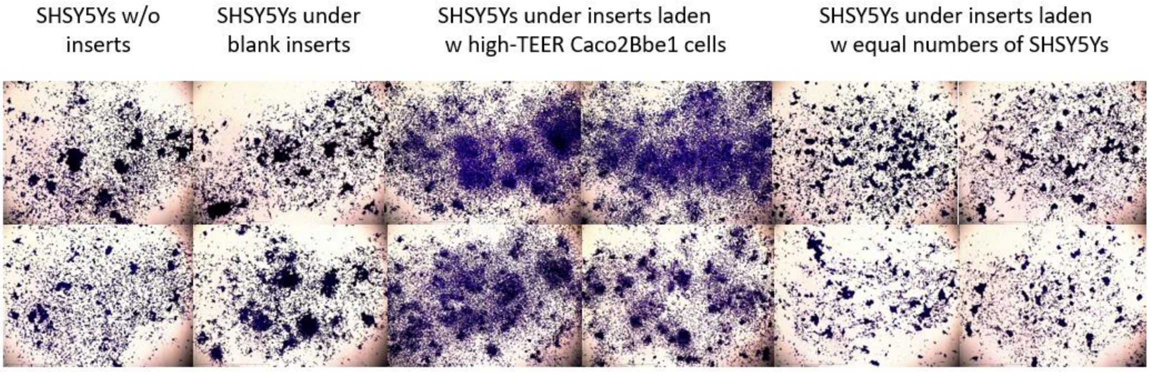

Figure S8. Microscopic view of anti-neuroblastoma model when maturated *Caco2Bbe1* inserts are co-cultured above near-confluent *SH-SY5Y's* remaining in 10% FBS media. This is the same experiment shown by eye in Figure S7. The *SH-SY5Y* cells had plated 5 days earlier with the same numbers per every well, differing only in treatments done the preceding 24 hours, as described in Figure S7. H&E staining was done synchronously in all wells and the microscope cameras settings were identical. The *SH-SY5Y* cells under *Caco2Bbe1* inserts were more dispersed with fewer punctate clusters. They also stained a different hue of crystal violet as shown in wells C3, C4, D3 and D4.

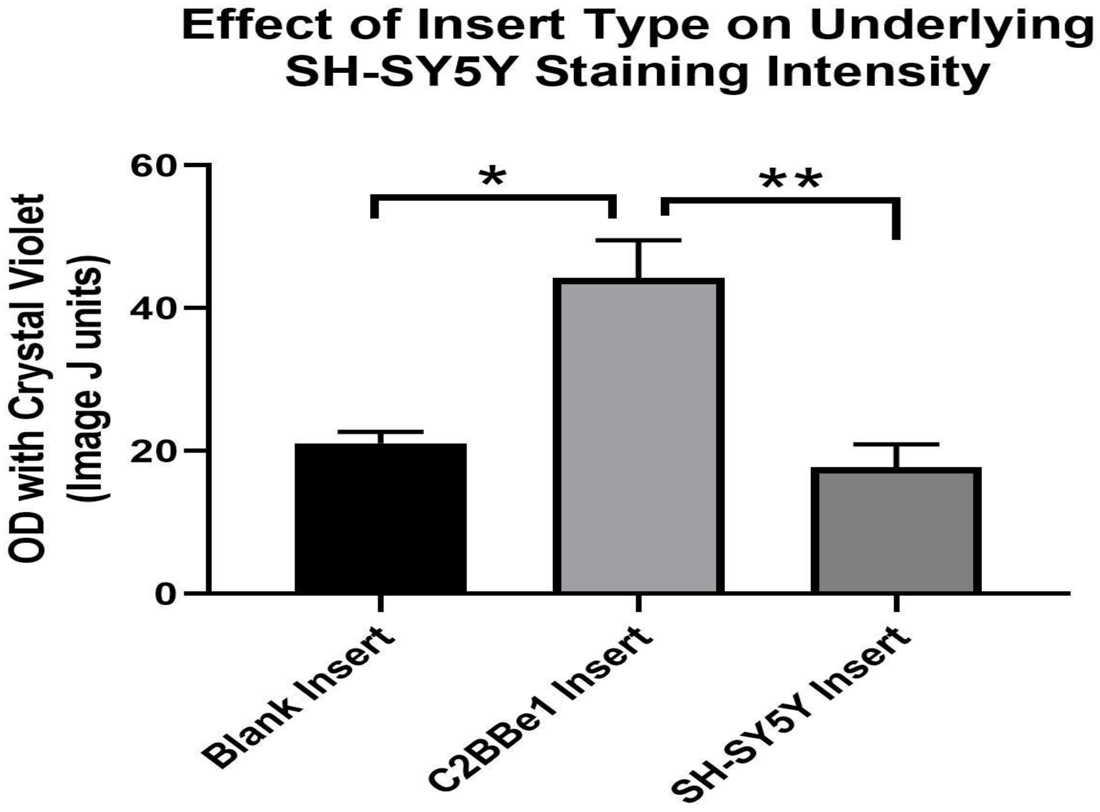

Figure S9. ImageJ results of alternative anti-neuroblastoma model when maturated *Caco2Bbe1* inserts are co-cultured above near-confluent *SH-SY5Y* cells remaining in 10% FBS media. This is the same experiment in Figures S7 and S8. The results could not be explained by more neuroblastoma growth under *Caco2Bbe1* inserts. Such an explanation would require the replication rate of the cells to be unbelievably fast in 24 hours. Rather, the *SH-SY5Y* cells exposed to *Caco2Bbe1* inserts changed from multiple compact clusters to dispersed neuroblastoma cells that covered the bottom of the dish. The higher staining is instead explained by a change in the phenotype of the neuroblastoma cells. Namely, they dispersed and took-up more stain in the dispersed state (Figures S7 and S8). Bars are means ± SEMs. The asterisks indicate high ($p < 0.05$) and higher ($p < 0.01$) significance (left to right).

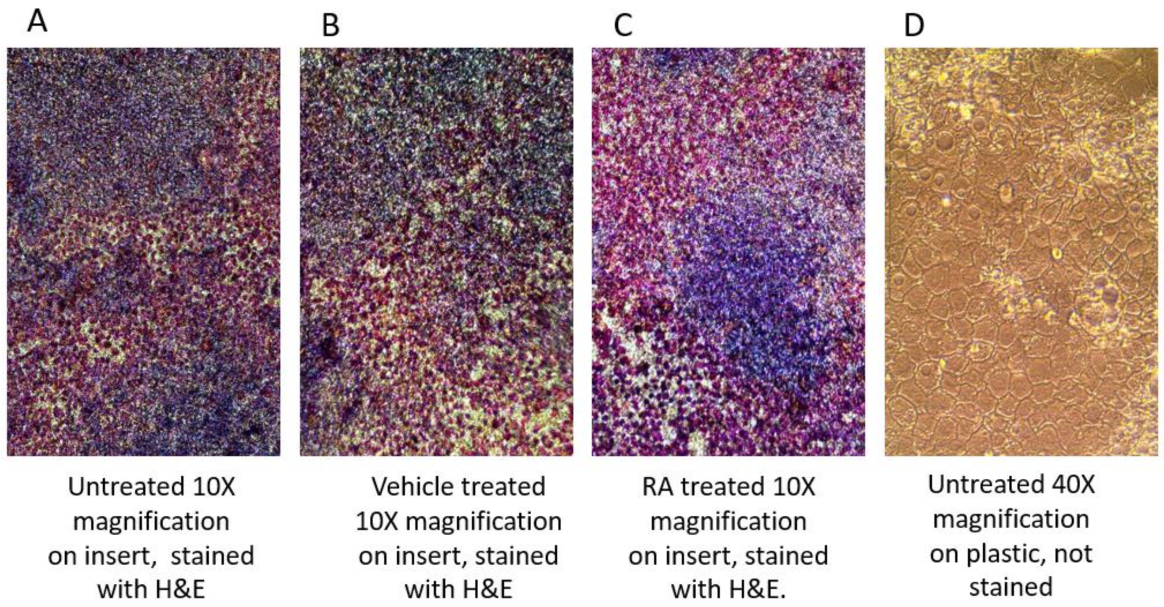

Figure S10. Microscopic comparison of mature *Caco2Bbe1* cells on day-21 in the fully developed model. All wells were plated from the same stock with identical numbers of cells and fed periodically as the model specifies in Figure S2. Cells in panels A-C were plated on filter inserts, treated as mentioned under the panel on day 19, and stained two days later with Hematoxylin and Eosin (H&E). The cells in panel D were plated in clear plastic wells and remained untreated until the image was captured at 40X magnification. Hematoxylin (H) binds nonspecifically to acidic components of cells, such as nuclei, appearing purple-blue color. All the cells showed the nuclei. Eosin (E) binds to basic components which are predominantly cytoplasmic, appearing pink-red color. According to previous studies, if abundant polyribosomes are present, H&E-stained cytoplasm appears a distinct blue cast. Golgi zones on the other hand can tentatively be identified by the absence of staining. Since the goldish color in panel D was imposed by the camera settings, we understand that this color represents unstained regions. And, to determine the nature of the unstained (goldish) regions in inserts in B-D, we super-imposed pre-stained and post-stained photographs. The unstained regions of the inserts in panels A-C were realized to correspond to large *Caco2-Bbe1* cells with spacious cytoplasm. The main experimental variable is found in panel C, the treatment with 10 micromolar retinoic acid (RA).
